# The dynamics of methylammonium ions in hybrid organic–inorganic perovskite solar cells

**DOI:** 10.1038/ncomms8124

**Published:** 2015-05-29

**Authors:** Aurelien M. A. Leguy, Jarvist Moore Frost, Andrew P. McMahon, Victoria Garcia Sakai, W. Kochelmann, ChunHung Law, Xiaoe Li, Fabrizia Foglia, Aron Walsh, Brian C. O'Regan, Jenny Nelson, João T. Cabral, Piers R. F. Barnes

**Affiliations:** 1 Department of Physics, Imperial College London, London SW7 2AZ, UK; 2 Department of Chemistry, University of Bath, Claverton Down, Bath BA2 7AY, UK; 3 Rutherford Appleton Laboratory, Harwell, Didcot OX11 0QX, UK; 4 Department of Chemistry, Imperial College London, London SW7 2AZ, UK; 5 Department of Chemical Engineering, Imperial College London, London SW7 2AZ, UK

## Abstract

Methylammonium lead iodide perovskite can make high-efficiency solar cells, which also show an unexplained photocurrent hysteresis dependent on the device-poling history. Here we report quasielastic neutron scattering measurements showing that dipolar CH_3_NH_3_^+^ ions reorientate between the faces, corners or edges of the pseudo-cubic lattice cages in CH_3_NH_3_PbI_3_ crystals with a room temperature residence time of ∼14 ps. Free rotation, π-flips and ionic diffusion are ruled out within a 1–200-ps time window. Monte Carlo simulations of interacting CH_3_NH_3_^+^ dipoles realigning within a 3D lattice suggest that the scattering measurements may be explained by the stabilization of CH_3_NH_3_^+^ in either antiferroelectric or ferroelectric domains. Collective realignment of CH_3_NH_3_^+^ to screen a device's built-in potential could reduce photovoltaic performance. However, we estimate the timescale for a domain wall to traverse a typical device to be ∼0.1–1 ms, faster than most observed hysteresis.

## Abstract (web summary)

Hysteresis often exists in the characterization of methylammonium lead halide-based solar cells, but is not well understood. Here, the authors use quasielastic neutron scattering to study the dynamics of dipolar organic cations and shed light on the hysteresis behaviour.

## Graphical abstract

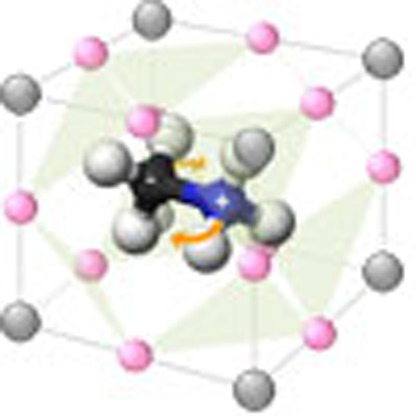

Hybrid organic–inorganic solar cells based on methylammonium lead halide perovskite structures are promising candidates for cheap, solution-processed, highly efficient solar cells with a short energy payback time^1^. Since the first publication in 2009 (ref. 2) on a solar cell made using this solution-processed material, the certified power conversion efficiency has now reached 20% (ref. 3). This article will focus on CH_3_NH_3_PbI_3_, which we will call MAPI, where MA^+^ refers to positively charged methylammonium ions, (CH_3_NH_3_)^+^. The perovskite crystal structure has an ABX_3_ stoichiometry coordinating like CaTiO_3_ (Fig. 1) where, in this case, A=CH_3_NH_3_^+^, B=Pb and X=I.

Perovskite structured compounds exhibit rich physical behaviour such as ferroelectricity, piezoelectricity, thermoelectricity, birefringence and superconductivity^4^,^5^,^6^,^7^,^8^. MAPI shows ambipolar charge transport and photo-generated charge diffusion lengths exceeding 100 nm (refs 9, 10). This allows the fabrication of devices in both nanostructured and planar heterojunction architectures with efficient charge collection (e.g., Chen *et al*.^11^ for MAPI). Reversible hysteresis is frequently observed in the photocurrent–voltage measurements used to characterize the efficiency of MAPI-based devices of all architectures^12^,^13^,^14^,^15^,^16^. Sweeping voltage from forward bias to short circuit results in higher measured photocurrents and open-circuit voltage values than sweeping in the opposite direction. In some cases, the photocurrent at a voltage equivalent to the maximum power point was shown to take ∼100 s to stabilize close to the value obtained from a forward to short-circuit sweep. The mechanism behind this phenomenon has not been established, but Snaith *et al*.^12^ suggested that it might be related to one of the following three processes: (a) the slow trapping or detrapping of charge in electronic interface states, further suggested in a recent paper by Bergmann *et al*^17^; (b) a slow ferroelectric polarization of the material dependent on applied bias; and (c) migration of excess ions as interstitial defects under the application of an applied bias; this could explain the photoinduced formation of iodide-rich domains in mixed-halide perovskites^18^, and might also explain the switchable photovoltaic effect observed in MAPI (although ferroelectric effects could also be responsible)^19^. A fourth process was suggested by Frost *et al*.^20^: migration of H^+^ formed by MA^+^ de-protonation during degradation.

Here we examine the possibility that the dynamics of organic cations (MA^+^ ions), which have an electrical dipole of 2.3 D (ref. 20) may contribute to the observed properties and behaviour of perovskite solar cells. The use of ferroelectric materials as light absorbers in photovoltaic (PV) devices has recently attracted attention^4^,^21^,^22^,^23^. Breaking the material's inversion symmetry results in spontaneous electric polarization that can enhance charge carrier separation relative to the unpolarized state, and in some cases can allow the photovoltage to exceed the bandgap by thousands of times^24^,^25^,^26^. However, the very weak poling of these ferroelectric materials under operating conditions relevant for solar cells together with their wide bandgap has limited their application in the field of PV until recently^4^,^27^. It was suggested that ferroelectric effects might exist in hybrid perovskites, since there are structural similarities with caesium plumbohalide and barium titanate crystals^28^. Since then, similar perovskite structures, such as CsGeCl_3_, have been reported as ferroelectrics^29^,^30^. More recently, remnant polarization of ≈10 mV has been reported for MAPI, which suggest a ferroelectric response^31^. Furthermore, Kutes *et al*.^32^ reported piezoforce microscopy measurements on 100-nm-thick MAPI films indicating the presence of ferroelectric domains (≈100 nm in size), which could be realigned on application of DC biases of 4 V or more.

Herein, we use quasielastic neutron scattering (QENS) measurements to directly measure the motions of MA^+^ ions within the inorganic lattice of the perovskite. The QENS technique is particularly sensitive to incoherent scattering events involving hydrogen nuclei (present at either end of the MA^+^ ions in MAPI), as the incoherent scattering cross-section of H is more than 20 times greater than that of the other elements in this perovskite^33^. Incoherent refers to the scattering from individual nuclei rather than coherent scattering, which gives information about interference phenomena between nuclei. A schematic of the QENS experiment is shown in Fig. 2, and a more complete description of the technique is given in the Methods section.

QENS neutron spectrometers typically probe motions in the ps–ns timescales and give insight into the self-correlation functions of H atoms to elucidate the spatial path and timescale of vibrational, rotational and translational diffusive processes at the molecular scale. We analyse the QENS data to demonstrate that the dipolar MA^+^ ions jump between preferential orientations within the surrounding PbI_3_ lattice and do not undergo free rotation (below 370 K), which would be characteristic of paraelectric rather than ferroelectric materials. Furthermore, no MA^+^ ion diffusion is observed within the experimental time window investigated (1–200 ps). The consequences of the discretized reorientation dynamics on the properties of the material and its relevance to photovoltaic devices are then assessed with Monte Carlo simulations of the MA^+^ realignment within the pseudo-cubic lattice.

## Results

### QENS measurements of MAPI

The dynamics of MA^+^ ions in the MAPI crystal lattice were probed using QENS. Representative quasielastic scattering spectra are plotted in Fig. 3a, these show the scattered intensity as a function of energy transferred (*ħω*) to or from the incident neutron beam owing to movement of the hydrogen nuclei within the sample. Spectra are shown for different momenta transfer, *Q*, where *Q* is determined from the scattering angle. The spectra are well modelled by a dynamic structure factor *S*(*Q*,*ω*) comprising two Lorentzian functions and an elastic peak, convoluted with the instrument resolution function (measured using a vanadium sample at 7 K), as illustrated in the inset of Fig. 3a. No additional modes of motion were required to describe the data (such as lattice modes involving atoms with small incoherent cross-sections relative to H, or modes occurring on a much longer timescale).

We now seek to identify the motions associated with each component of quasielastic broadening in the scattering spectra. Figure 3b shows the full-width half-maximum (FWHM) of each Lorentzian as a function of momentum transfer squared (*Q*^2^) plotted for a range of temperatures. The width of both Lorentzians remains approximately invariant with *Q*^2^, within experimental uncertainty, at all temperatures investigated. This is consistent with localized motions, in particular rotational or reorientation motions of the MA^+^ ions, and rules out diffusive translational motion below 370 K, for which the FWHM would increase linearly with *Q*^2^ for Fickian diffusion^34^,^35^. Our data are incompatible with MA^+^ diffusion confined within a spherical cavity of radius commensurate with the perovskite cage (Supplementary Fig. 1; refs 36, 37). Thus, we conclude that the MA^+^ ions can undergo rotation movements constrained inside the inorganic cages within the range of the measurement.

Recent experimental evidence indicates the possible presence of several weight per cent remnant solvent, dimethylformamide (DMF) trapped within crystalline samples following preparation^38^. Our measurements suggest that up to ∼2% of our measured sample weight could be residual gamma-butyrolactone (GBL) entrapped in the material (Supplementary Fig. 2). Upon inspection of X-ray diffraction and Fourier transform infrared spectroscopy data collected during sample preparation (Supplementary Figs 3 and 4), as well as neutron diffraction data from the material combined with the temperature dependence of the effective Debye–Waller factor (Supplementary Figs 5–7), we find no evidence that trace quantities of GBL influence our analysis of MA^+^ motion, which we take to indicate that, if present in our sample, its dynamics fall outside the spatio-temporal window probed. However, ∼13 % uncertainty is added to the evaluation of the fraction of rotationally active of scatters in the material (Supplementary Fig. 2).

**Figure 1:**
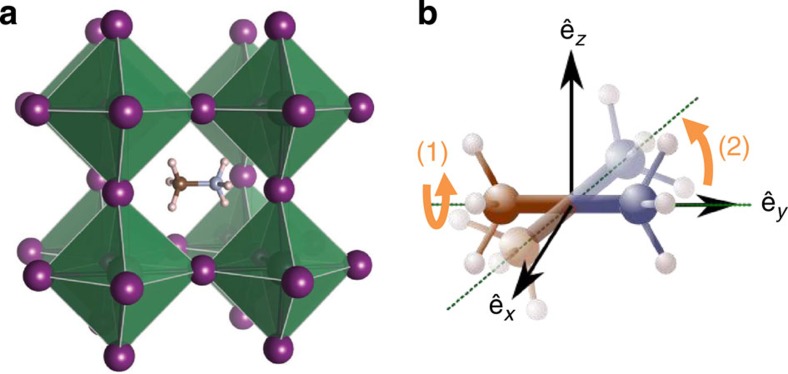
CH_3_NH_3_PbI_3_ crystal structure and CH_3_NH_3_ rotational modes. (**a**) Crystalline structure of MAPI in the cubic (*T*>330 K) phase. A methylammonium molecule occupies the A-site enclosed by octahedra; iodide atoms form the vertices of the octahedra (X-sites, depicted in purple), while the lead atoms occupy their centres (B-sites); from Brivio *et al*.^63^. (**b**) Schematic of two reorientations: (1) methyl and/or ammonium rotation around the C–N axis (the carbon and nitrogen atoms are depicted in blue and brown, respectively, the axis is indicated by the green dotted lines); (2) whole molecule reorientation via rotation of the C–N axis itself.

Based on the analysis of the elastic incoherent structure factor (EISF; discussed below), we assign the wider Lorentzian ‘1' function to rotation of the methyl and/or ammonium groups around the C–N axis of the MA^+^ and the narrower Lorentzian ‘2' to the tumbling of the C–N axis within the cage (Fig. 1). We also note that no major change occurs through the crystal phase transitions of MAPI known to occur at 162 and 327 K (ref. 39), despite minor structural changes in the neutron diffraction patterns being observed across these temperature thresholds (Supplementary Figs 6 and 7).

### Analysis of MA^+^ reorientations in MAPI

The most stable configuration of the MA^+^ ion and the degeneracy of its orientational states has been the subject of considerable debate^40^,^41^,^42^,^43^,^44^,^45^,^46^. To examine the geometry of the rotational motions inferred from Fig. 3b in further detail, we computed the ratio of elastically-to-elastically and quasielastically scattered neutrons from the scattering spectra as a function of the momentum transfer, *R*_*i*_(*Q*)=*Λ*_E_(*Q*)/[*Λ*_E_(*Q*)+*Λ*_*i*_(*Q*)]. *R*_*i*_ was determined for each motion independently for each of the two fitted Lorentzians, *i*=1, 2, where *Λ*_E_ is the area under the elastic peak and *Λ*_*i*_ is the area under the corresponding Lorentzian. This ratio was fit with an expression containing the EISF, which is a temperature-independent expression for elastic scattering intensity. The functional form of the EISF is defined only by the geometrical path explored by scatterer (that is, the hydrogen nuclei) within the time window of the instrumental range. The likelihood of possible modes of rotation or movement can be assessed by comparing fits using different theoretical EISFs to the *R*_*i*_(*Q*) data (Fig. 4). Details of the model fitted and different EISF functions are in the Methods section and Supplementary Table 1.

The ratio *R*_1_(*Q*) for the scattering component assigned to the rotation of H nuclei around the C–N axis (mode ‘1' in Fig. 1b corresponding to the broad Lorentzian in Fig. 3) is best described by either free rotation of the hydrogens in methyl and/or ammonia about the C–N axis (Supplementary Fig. 8) or by the ‘clicking' (that is, activated rotation) of the hydrogens between sites of threefold symmetry^47^,^48^. These motions have a similar geometry that could not be distinguished within experimental uncertainty (Supplementary Fig. 9 and Supplementary Table 1)^49^.

To describe the realignment of the mobile fraction of MA^+^ (mode ‘2' in Fig. 1b corresponding to the narrow Lorentzian in Fig. 3), we evaluated a number of possible tumbling mode models. Some of the motions considered are illustrated in Fig. 4a. Further details of models, molecular trajectories and detector group choice are presented in Supplementary Figs 10–13 and Supplementary Table 1.

Figure 4b shows the elastic-to-elastic and quasielastic scattering ratio attributed to C–N reorientation plotted as a function of *Q*, (*R*_2_(*Q*) corresponding to the narrow Lorentzian in Fig. 3, mode ‘2'). Interestingly, the shape of this ratio as a function of *Q* was independent of temperature (Supplementary Figs 10 and 11), this was also the case for rotation mode ‘1' (Supplementary Figs 8 and 11). This suggests that there is no significant change in the type of rotational motion through the crystalline phase changes at 162 and 327 K. Also shown in Fig. 4b are the theoretical models using different EISFs corresponding to motions (i), (ii), (iii), (iv) and (v), the functional forms are given in Supplementary Table 1. It is apparent that the models for free tumbling (i) that would be consistent with a paraelectric material and π-flips (ii) of the MA^+^ ions are inconsistent with the data, and are thus unlikely to occur on the timescales examined (1.2–53 ps). Conversely, models (iii–v) show reasonably good agreement with the data, all three involve conical particle trajectories. While we cannot unambiguously distinguish between these three modes, we can conclude that realignment of the MA^+^ ions between either corners, edges or faces are likely to occur within the cages. Previous studies inferred that MA^+^ ion alignment towards cage corners^45^ or edges^41^,^43^ were the most energetically stable/likely configuration. Results from recent *ab initio* molecular dynamic (MD) calculations, which assume that the system is ergodic^40^, are presented in Fig. 4c. These suggest that the preferred orientation of the MA^+^ ions is towards the cubic faces, which is consistent with model (iv).

The average residence time for a given MA^+^ alignment (*τ*_2_) was estimated from 2*ħ*/FWMH using the FWHM derived from all detector *Q* groups in Fig. 3b. At room temperature, this corresponds to *τ*_2_=14±3 ps (see Supplementary Table 2 for other temperatures), which is somewhat longer than previously estimated from nuclear magnetic resonance measurements (0.2 ps), and from millimetre-wave spectroscopy (5.37 ps; refs 45, 50). For comparison, the mean residence time inferred from the MD calculations in ref. 40 is 3.14 ps and in ref. 51 is 4–6 ps, which are also shorter but remain of the same order of magnitude as our observed values. Arrhenius activation energies were also estimated from the FWHMs and found to be 9.9 meV for rotation 1 within the interval of confidence given by the external slopes: [0; 18.5], and 13.5 meV for rotation 2, within [1.2; 24.2] (Supplementary Fig. 14 and the corresponding residence times in Supplementary Table 2). This is consistent with the typical activation energies of methyl rotations (a few tens of meV (ref. 52)) and compares with previously reported values for MAPI of 52 meV for rotations about C–N^50^. The values of 117 (ref. 51) or 101 meV (ref. 46) for reorientations of the axis itself has been estimated for MAPI, and 126 meV for Cl and Br perovskites, respectively^50^. Calculated values of the energy barrier for ion rotation determined from *ab initio* modelling indicated values of 13.5 meV (in agreement with our observations)^20^, whereas the value for jump diffusion of MA^+^ is possibly as high an eV if not mediated by defects.

In addition to the increase of reorientation rate with temperature (Supplementary Table 2), our analysis also allows us to determine the fraction of molecules involved in reorientation within the measurement timescale. This fraction is determined from the only fitting parameter in addition to the choice of EISF used to describe the ratio of elastic-to-quasielastic scattering intensity data (for example, Fig. 4b) as described in the ‘Analysis of rotational movements' section of the Methods). The results in Fig. 4d indicate that the fraction of methyl and/or ammonium rotors involved in rotations (mode ‘1') steadily increases with temperature. This behaviour with temperature could be consistent with a temperature-activated cleaving of ‘hydrogen bonds' between the hydrogens and the surrounding iodine atoms as suggested by DeAngelis and colleagues^49^ Alternatively, it could be related to a reduction in steric hindrance as temperature increases since the material has been reported to be very soft on an atomic scale, with atoms exploring the unit cell very far from their equilibrium position^53^. QENS experiments using partially deuterated MA^+^ ions could help resolve whether one end of the MA^+^ ions is bound within the cage although exchange of H an D nuclei might prove problematic.

Figure 4d also indicates only ∼20 % (ranging between 17 and 35 %) of the hydrogen nuclei in MA^+^ ions being involved in realignment of the C–N axis within the experimental time window (this changes to 23–40% if accounting the possibility of entrapped solvent ions contributing to the elastic signal), and that this fraction is relatively independent of temperature. This observation suggests that the rotating fraction could correspond to the proportion of MA^+^ located in cages in the vicinity of crystal grain boundaries or surfaces, since these areas would not change with temperature. Related to this explanation is the possibility that the MA^+^ ions with orientation residence times exceeding 200 ps (≈60–77%) are stabilized within ferroelectric domains. If this hypothesis is correct, then the interaction of the MA^+^ dipole with the surrounding lattice and neighbouring dipoles could result in a locally preferred alignment relative to the surrounding ions. We note that the realignment of the MA^+^ ions is likely to be strongly coupled to deformation of the surrounding I and Pb atoms in the lattice, although it is not clear which movement is causative^51^,^53^, this will also contribute to the magnitude of polarization.

### Monte Carlo simulation of interacting MA^+^ dipoles

To investigate whether ferroelectric domains are formed, we performed Monte Carlo simulations considering a three-dimensional (3D) lattice of dipoles that may reorientate to align with the faces, corners or edges of the pseudo-cubic cage (26 possible alignments), which is consistent with the EISF analysis in Fig. 4b. The simulation accounts for the dipole–dipole interaction energies in the lattice, as well as incorporating the effect of cage deformation (strain) resulting from rotating the MA^+^ ion within a lattice on energy of site. The magnitude of both dipole–dipole interaction energy and the strain term energy were determined from *ab initio* calculations. The energy of the strain term was estimated (*K*=25 meV) by rotating the MA^+^ ion through 180° within a lattice in which all other MA^+^ ions were aligned in the same direction towards the face of a cube and calculating the change in energy of the system after subtracting the energy change due to dipole–dipole interactions. Owing to the uncertainty in both the lattice screening of the dipole–dipole interactions and the strain term that compete against each other, two examples of simulations are presented (*K*=25 and 100 meV) that illustrate the different general types of behaviour observed.

As the temperature was reduced, the dipoles began to spontaneously form either antiferroelectric or ferroelectric domains dependent on the relative magnitudes of dipole and strain terms (Supplementary Figs 16–24). Results of the simulations cooled to 50 K (chosen to display the structures with minimal thermal noise relative to higher temperatures) are shown in Fig. 5. In the *K*=25 meV case, the dipoles formed a complex arrangement of canted ‘head to tail' twinned layers antiparallel to neighbouring pairs of layers (see Fig. 4a,c and Supplementary Fig. 16 for orientations during cooling). This corresponds to an antiferroelectric arrangement in which no net electric field is present within a domain. Our previous 2D calculations suggested antiferroelectric behaviour for *K*=0 (ref. 53). Herein, at 300 K, we observe that considerable thermal noise is superimposed on the antiferroelectric structure (Supplementary Fig. 16).

Ferroelectric domains in which all dipoles had parallel alignment formed when *K* was greater than ∼50 meV; see Fig. 4b,d and Supplementary Fig. 20 for the *K*=100 meV case. In this example, ferroelectric arrangements formed even at temperatures >350 K and electric fields were induced across the domains owing to the net displacement of charge to opposite domain walls. If domains are small and randomly aligned relative to the MAPI film thickness, then no net electric field is expected across the film since the E-field of numerous domains would average to zero. However, if ferroelectric domains in MAPI films span the entire film as suggested recently^32^, then we can expect an electric field to be induced spanning the device. Note that, although our results only explicitly show the alignment of the MA^+^ dipoles, they implicitly include the electrostatic effects owing to the displacement of the other atoms within the lattice, which will scale the results linearly. Thus, the behaviours simulated here should still be relevant to hybrid perovskite structures containing cations with weaker dipoles than MA^+^ ions.

Figure 4e,f indicates that MA^+^ ions in the vicinity of domain boundaries are more likely to undergo realignments than those stabilized in the bulk of domains leading to a multimodal distribution of realignment probabilities (Supplementary Figs 19 and 23 show the results for all temperatures), suggesting the possibility that only the MA^+^ ions near domain walls underwent reorientation on the timescale of the QENS measurement. This hypothesis could explain the temperature-independent population of immobile MA^+^ ions observed in the QENS measurements (Fig. 4d) if the density of domain walls/grain boundaries is temperature independent. The simulated proportion of MA^+^ in stable orientations corresponding to ferroelectric domains was only weakly dependent on temperature below 350 K (Supplementary Fig. 23). We note that in MAPI films, the formation of domains is likely to depend on the details of the material preparation and cooling conditions, as well as being strongly influenced by the presence of mesoporous scaffolds, so the fraction of MA^+^ at domain walls would be a sample-dependent parameter.

## Discussion

We now discuss the possible implications of antiferroelectric or ferroelectric domain formation for device behaviour. Figure 5g,h shows the sections of the local electrostatic potential resulting from the simulated dipole orientations. In both the examples, heterogeneity is indicated and is associated with the domain walls. The magnitude of the heterogeneity is much greater in the ferroelectric arrangement (Fig. 5b). Note that the calculated domain sizes, and thus the length scale of the heterogeneity in the lattice, depend on the details of the simulation protocol and could be much larger if smaller temperature steps were used during the cooling. The domain sizes observed experimentally were on the order of 100 nm (ref. 32). Heterogeneity (Fig. 5h) in the electrostatic potential of the lattice could result in spatial separation of free electrons and holes to opposite domain walls to minimize their free energy. This requires that domain sizes are larger than the polaron radii (∼5 unit cell sides, ∼32 Å; ref. 53). Recent calculations on supercells of static, randomly aligned MA^+^ ions indicated that even random disorder could result in an increase in charge carrier lifetimes owing to localized covariation of the conduction and valence band edges^54^. Consequently, heterogeneity in the potential could extend the lifetime of the carriers beyond that in a homogeneous material and might contribute to the long-carrier collection lengths that have been observed^9^,^10^,^55^.

E-fields typical of working devices (≤5 MV m^−1^ for a 200-nm-thick film) result in a drop of <3 mV per unit cell. This is significantly less than the magnitude of the strain and dipole–dipole energies stabilizing the MA^+^ orientations so that field-induced reorientation is most likely to occur at domain walls where the MA^+^ orientation is less stable (Fig. 5e,f). Movement of domain walls perpendicular to the field is expected for antiferroelectric domains in the presence of an E-field. Although the consequences of this on device performance are not obvious, a field between the contacts could result in potential barriers parallel to the plane of the contacts or reduced heterogeneity, which might inhibit charge collection. Varying E-field may also result in changes in the dominant domain wall type. Different domain walls (90° versus 180°) have been calculated to result in changes in charge collection efficiency^56^. A further possibility is that domain wall movement might influence recrystallization of physical grain boundaries.

If ferroelectric behaviour is present, the built-in E-field at short circuit resulting from the difference in contact work functions would eventually induce alignment of ferroelectric domains between the contacts, which would screen the built-in potential. The polarization of a ferroelectric domain boundary in this model, accounting only for the MA^+^ ions, is *P*≈3 μC cm^−2^, the polarization for the whole electronic lattice was previously calculated to be *P*=38 μC cm^−2^ (ref. 20). The MA^+^ ions alone could result in several hundred mV of reduction in the built-in potential depending on the dielectric constant (*P* × thickness/(*ɛ*_0_*ɛ*_r_)). With this screening, a lower E-field is available to separate charge carriers at short circuit, and a lower open-circuit potential would be achievable under the polarized conditions. However, if the device is held under forward bias or sustained open-circuit illumination conditions, the absence of a strong electric field would allow the random relaxation of domain alignment, removing the screening effect. Returning the device to short circuit from this state would result in the full built-in potential drop across the photoactive layer. Under these randomized conditions, more efficient charge collection and a higher open-circuit potential would be achieved before the domains are realigned under the built-in potential (Fig. 6). We note that a similar picture of device behaviour and polarization would occur due to the drift of ions through defects in the lattice.

We can estimate a lower limit for the timescale needed for the reversal in the polarization of a ferroelectric domain spanning a device. By combining our experimental and modelling results the time for a domain wall to cross a MAPI film for the simulated ferroelectric example could be approximated. Assuming that the orientation residence time (*τ*_2_) inferred from the neutron scattering measurements corresponds to MA^+^ ions at domain walls, then the attempted move rate is given by *k*=*P*_DW_/*τ*_2_, where *P*_DW_ is the probability of an attempted reorientation being successful in the vicinity of a domain wall. At room temperature, *k*≈10^9^ s^−1^ using *τ*_2_=14 ps and *P*_DW_∼0.015 (inferred from the geometric mean of the domain wall peaks in the histogram in Supplementary Fig. 23). Applying a 5-MV m^−1^ E-field to the simulation volume results in a change in the probability of *P*_DW_ by Δ*P*_DW_ (Supplementary Fig. 26). This change corresponds to the probability that a rotational move will be influenced by the E-field rather than random. Thus, the upper limit of the instantaneous domain wall velocity on application of an E-field is roughly given by *v*_DW_≈*k* Δ*P*_DW_ *L*, where *L* is the unit cell dimension. At room temperature, *v*_DW_≈0.3–3 mm s^−1^ (using Δ*P*_DW_≈10^−4^–10^−3^ and *L*=6.3 Å). At this initial speed, a domain could cross the thickness of a 200-nm device in ∼0.1–1 ms. The hysteresis observed in MAPI devices occurs over a range of timescales extending to >10 s (refs 12, 13, 14, 15), which is significant longer than our upper limit for the transit time. Thus, it appears unlikely that simple ferroelectric effects could be responsible for the hysteresis that has been observed in devices on longer timescales, unless the domain structures are considerably more stable than we have estimated here. However, our estimate neglects collective effects and the relaxation of domain wall velocities, which have been observed to slow down after the onset of an E-field^57^. We also note that if lateral domain growth controls hysteresis, then domain alignment times could be substantially longer. Recent small perturbation photovoltage transient measurements of MAPI devices have shown biphasic photovoltage decays, where the slow phase, which is not fully understood at present, has a time constant in the range 1–100 ms (ref. 58), which also could be consistent with the timescale estimated here.

In summary, we have presented direct evidence for the motion of methylammonium ions within CH_3_NH_3_PbI_3_ using QENS. Analysis of the dynamic structure factor indicates the rotation of CH_3_ and/or NH_3_ groups around the C–N axis of the MA^+^ ions, and also the rotational realignment of the C–N axis itself within the cages formed between the PbI_3_ octahedra of the surrounding lattice. This realignment is consistent with jumps between the faces, corners or edges of the surrounding cage taking ∼14 ps at room temperature. No evidence was observed for the translation of the MA^+^ ions between cages within the time window (1.2–53 ps). In this polycrystalline sample, 20–40% of the MA^+^ underwent rotations within the time window independently of the temperature observed (140–370 K). With the aid of Monte Carlo simulations of dipole–dipole interaction between MA^+^ ions and lattice deformation, we have shown that this observation could be consistent with C–N reorientation only occurring in the vicinity of boundaries between antiferroelectric or ferroelectric domains. If antiferroelectric domains form at room temperature, they are not expected to significantly influence charge carrier behaviour since the fields from the neighbouring dipoles cancel. However, ferroelectric domains may form if dipole interactions are screened by the inorganic lattice, or where the energetic penalty for lattice deformation by C–N rotation is greater than estimated. Ferroelectric domains could help separate free electrons from holes; however, the domains would also screen the built-in potential within devices, resulting in reduced charge collection efficiency and initially low open-circuit voltages. Relaxation of the dipole alignment in the absence of significant field (open circuit or forward bias conditions) would lead to higher charge collection efficiency. A crude estimate for a timescale of ferroelectric domain relaxation within a device based on our observations is roughly 0.1–1 ms. Many observations of photovoltaic hysteresis in CH_3_NH_3_PbI_3_ devices are greater than this value^16^, suggesting that other effects such as ionic diffusion may also play a role.

Ferroelectric domain alignment can be induced by engineering strain or permanent dipoles at crystal interfaces^59^. If ferroelectric effects influence the behaviour of MAPI devices, then careful choice of the contact materials to align domains to enhance rather than screen the built-in potential will be key to optimizing performance and stability.

## Methods

### Sample preparation

The detailed method to synthesize CH_3_NH_3_I from concentrated aqueous hydroiodic acid and methylamine can be found in ref. 60. Approximately 1 g of MAPI was required to give the optimal scattering signal for the neutron measurements. This was prepared from a solution containing an equimolar mixture of methylammoniumiodide and lead iodide (PbI_2_) in 1.25 mol l^−1^ GBL. The solution was directly deposited on a layer over aluminium foil (area ≈300 cm^2^) on a hot plate at 100 °C in air. After 2 h, the yellow solution had crystallized into a dark solid layer of MAPI, which had reached a stable mass (to within 2%; Supplementary Figs 2–4). The crystalline layer on aluminium was then folded into a rectangle (5 × 7) cm and wrapped in an annulus and sealed within an aluminium annular cylindrical sample can of 2 mm gap. The neutron diffraction spectrum of the sample collected using GEM^61^ indicated that MAPI was formed (Supplementary Fig. 5).

### Quasielastic neutron scattering

**Figure 2:**
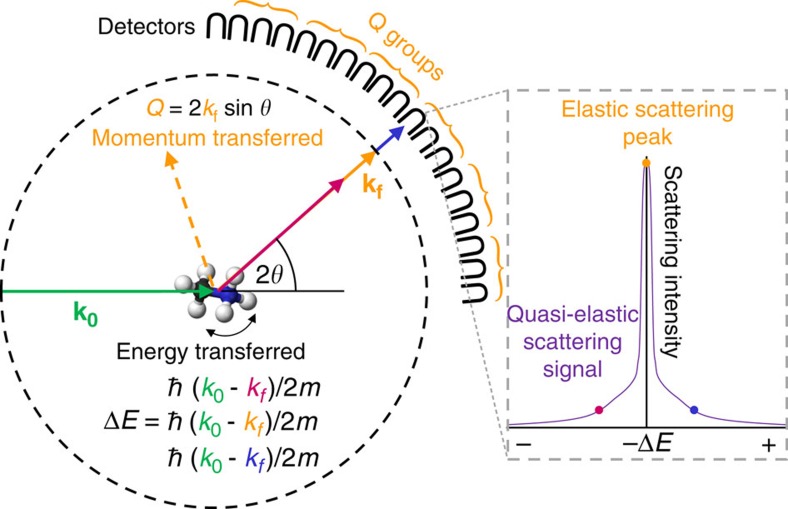
QENS setup. The momentum of the incident (**k**_**0**_) and scattered (**k**_**f**_) neutrons, the corresponding (elastic) change in momentum (*Q*), which is related to the scattering angle (2*θ*), and the energy transferred in the quasielastic scattering event (Δ*E*) are indicated. Measurement statistics can be improved by averaging data from groups of detectors (*Q* groups). A schematic quasielastic scattering spectrum is indicated from one of the detectors, part of this signal is elastic (that is, Δ*E*=0). While OSIRIS operates with a backscattering detector geometry, for simplicity, the neutron detectors are shown in forward direction. An initial neutron energy band is selected and the final neutron energy analysed by an array of analyser crystals before detection by the neutron detectors.

A schematic indicating principles of QENS is shown in Fig. 2, the sample is exposed to a beam of thermal energy neutrons (*E*=*ħk*_0_/2*m* (in this case ∼0.002 eV), where *m* is the mass of the neutron). The energies and angles of neutrons scattered from the sample are measured with an array of detectors enabling the change in momentum (*Q*) and change in energy (Δ*E*=*ħω*, where *ω* is the change in angular frequency of the scattered neutron) to be determined. The resulting quasielastic scattering spectra determined for different *Q*s and temperatures can then be analysed to resolve the geometry and frequency of motions occurring within the experimental spatio-temporal window. Elastic scattering events involve no energy exchange between the neutron and the sample (Δ*E*=0) together with events involving an exchange of energy smaller than the resolution of the spectrometer, which appear as an elastic peak in the spectrum. Atomic motions in the sample, occurring within the energy window of the spectrometer, can result in the scattered neutron gaining or losing energy (Δ*E*≠0). The extent to which this occurs depends on both the rate of the movement in the sample and the geometrical path that the scatter moves through relative to the change in momentum in the scattering event (determined by the scattering angle). The measured scattering intensity is typically a broad peak, whose breadth is proportional to the timescale of the motion, centred around the elastic peak at Δ*E*=0.

The QENS experiments were conducted with the high flux time of flight spectrometer OSIRIS (ISIS-pulsed neutron source, Rutherford Appleton Laboratory, UK). In this setup, the energy of scattered neutrons is analysed by Bragg scattering from a PG002 crystal analyser array (*E*_f_=1.84 meV) combined with 42 ^3^He detectors^62^. The instrument incident energy is set to measure energy transfers between −0.55 and 0.55 meV energy transfer with a resolution of 25.4 μeV, corresponding to the minimum detectable FWHM. The *Q*-range investigated varied from 0.18 to 1.8 Å^−1^, while sample temperature was increased from 7 to 400 K. The beam intensity at the sample is 2.7 × 10^7^ neutrons cm^−2^ s^−1^ at 150 μA ISIS beam current. Low statistic measurements (15 μA h) were made at 10 K steps between 7 and 400 K. High statistic measurements (300 μA h) were conducted at 7, 40, 140, 180, 250, 300 and 370 K to provide reliable data to investigate MA^+^ dynamics (corresponding to ∼2 h measurement each, since additional time is required to stabilize the temperature in the cryostat). Further details on the interpretation of the measured data are given in Supplementary Note 1.

### Analysis of rotational movements

Under the assumption that the signal collected on OSIRIS (*cf*. Supplementary Information) is solely caused by incoherent scattering events arising from the hydrogen nuclei, the incoherent scattering law assuming two independent rotational reorientations can be expressed as:


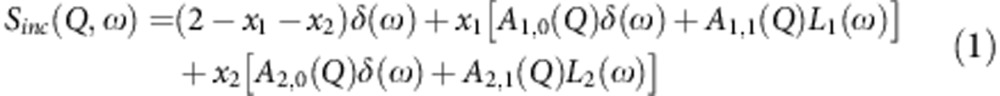


where *δ*(*ω*) is a delta function, *x*_*i*_ is the fraction of scatterers involved in the *ith* rotational mode (*i*=1 or 2), *A*_*i*,0_(*Q*) and *A*_*i,1*_(*Q*) are proportional to the EISF and the first term of the quasielastic incoherent structure factors, respectively, for the corresponding rotational mode *i* (Supplementary Note 2), and *L*_*i*_(*ω*) is a Lorentzian function defined by:


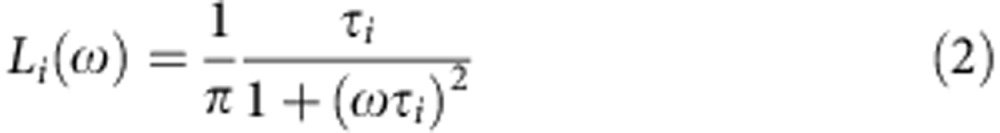


where *τ*_*i*_ is the correlation (residence) time for the first-order spherical harmonic for motion *i*, which is related to the energy transferred (Δ*E*=*ħω*) to the scattered neutrons by *τ*_*i*_=2*ħ*/FWHM_*i*_(Δ*E*). In the expression for *S*_inc_(*Q*,*ω*), equation (1), the scattering function for each rotational mode has been split into a purely elastic component *A*_*i,0*_(*Q*)*δ*(*ω*) superimposed with a quasielastic broadening function, *A*_*i*,1_(*Q*)*L*_*i*_(*ω*) where the sum of these two terms is 1; higher-order rotational harmonics are assumed to give a negligible scattering contribution.

**Figure 3:**
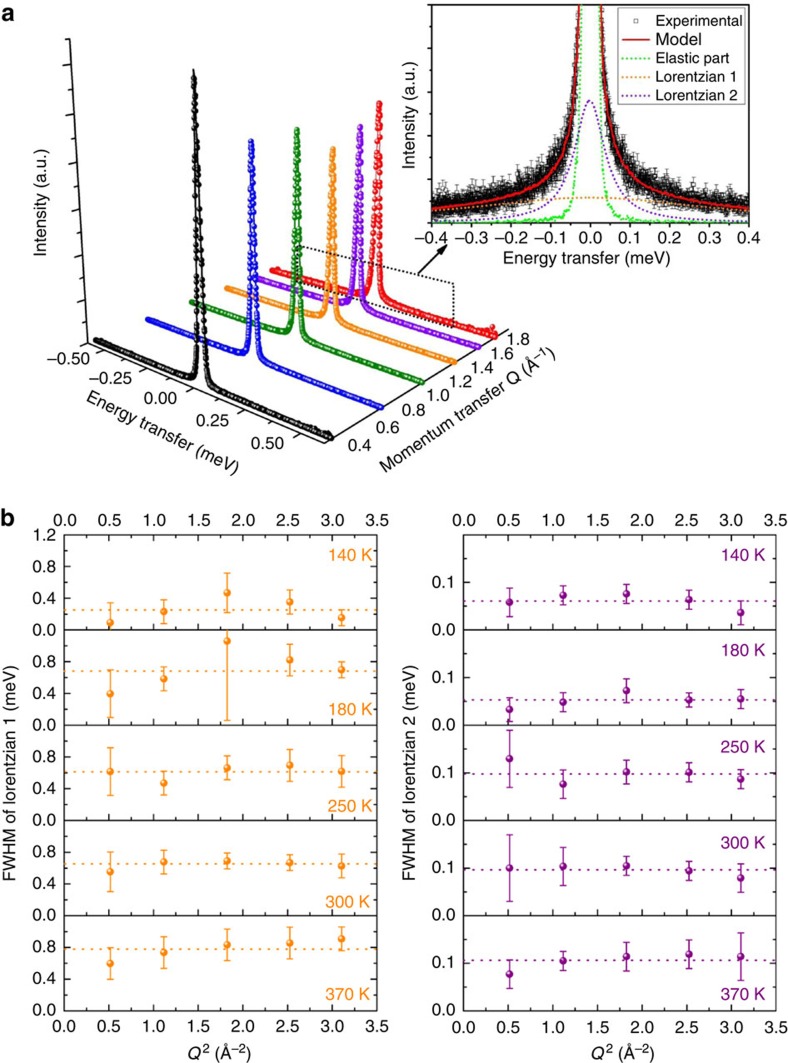
QENS data collected from MAPI. (**a**) Representative QENS measurements indicating scattered intensity against energy transfer, *ħω* (eV), measured at 300 K plotted for groups of seven detectors corresponding to different momentum transfer, *Q* (Å^−1^). Inset: the spectrum corresponding to *Q*=1.76 Å^−1^. The experimental data are represented by hollow squares with associated error bars. The solid red curve corresponds to the fit of *S*(*Q*,*ω*) consisting of the convolution of the instrument resolution with an elastic peak (dotted green line, which encloses area *Λ*_E_), and two Lorentzians (dotted orange and violet lines enclosing areas *Λ*_1_ and *Λ*_2_). (**b**) FWHM of the two Lorentzian functions used to fit the QENS data as a function of momentum transfer squared for different temperatures. The dotted lines show the average FWHM. A.u., arbitrary unit.

**Figure 4:**
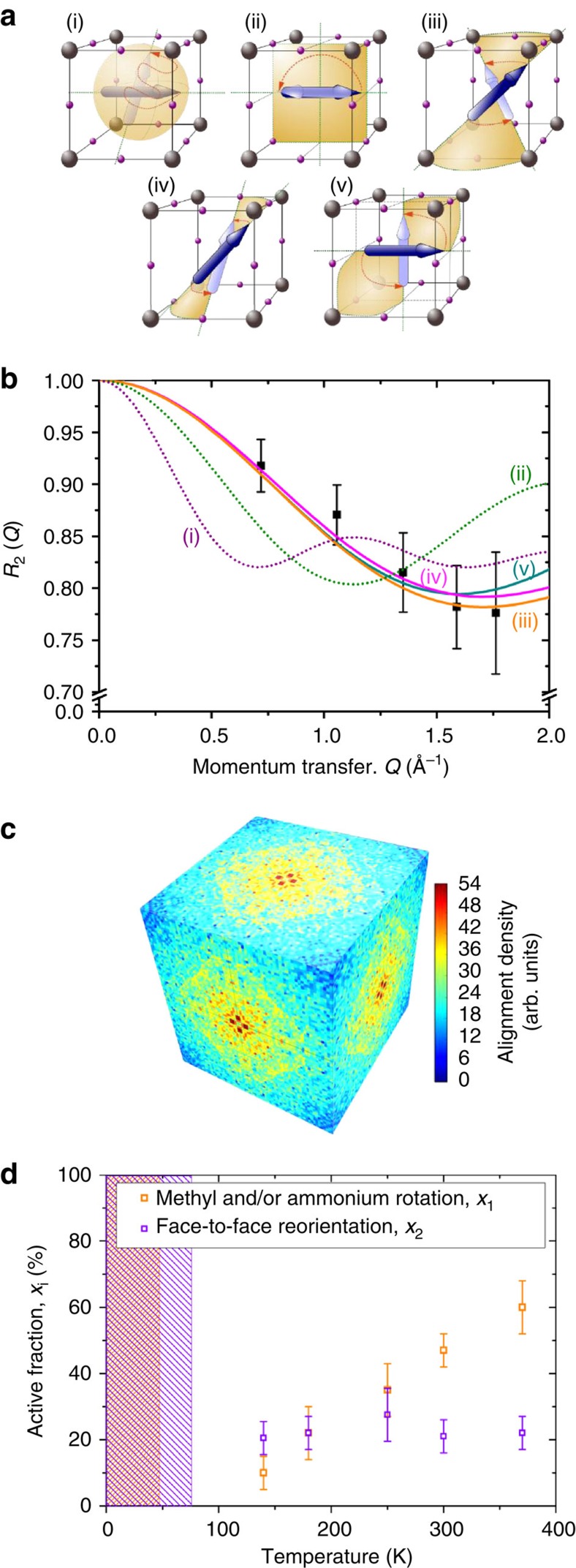
Analysis of MA^+^ reorientation within MAPI. (**a**) Possible reorientation pathways for MA^+^ ions (mode ‘2') within a PbI_3_ cage: (i) free rotation of MA^+^ ions in the inorganic cage, this mode would be characteristic of a paraelectric material with no significant residence time in any specific direction. (ii) π-Flips, which might be expected for constrained dipole reorientations in ferroelectric or antiferroelectric materials. (iii) Corner-to-corner hops, where the MA^+^ ion alignment reorientates from one diagonal to another neighbouring diagonal (<111> orientations). (iv) Edge-to-edge hops, involving the MA^+^ ion's reorientation from one edge of the cubic cage to another neighbouring edge (<110> orientations). (v) Face-to-face hops, in which the MA^+^ ion reorientates from an alignment pointing towards a face of the cubic cage to facing a neighbouring face (<100> orientations). (**b**) Representative ratio of elastic-to-elastic and quasielastic scattering, *R*_2_=*Λ*_E_/(*Λ*_E_+*Λ*_2_), assigned to C–N axis reorientation in MAPI (that is, mode ‘2') at 370 K plotted against *Q*. Also shown are the fits incorporating the theoretical EISFs for the different motions depicted in **a**, accounting for the active fraction given in **d**. (**c**) Distribution of MA^+^ ion alignments calculated from *ab initio* quantum mechanical MD modelling based on calculations in ref. 53 projected onto the sides of a lattice cage where the corners would be occupied by Pb atoms. (**d**) The fraction of MA^+^ ions involved in each type of dynamic rotation within the time window of the measurement. The rotations correspond to either CH_3_ and/or NH_3_ rotors (broad Lorentzian, mode ‘1', orange), or tumbling of the C–N axis (narrow Lorentizian, mode ‘2', purple). The shading indicates the temperature ranges in which the populations of threefold rotation of CH_3_ and/or NH_3_ (orange) or C–N axis reorientation (purple) could not be fully resolved by the QENS spectrometer (Supplementary Fig. 15).

**Figure 5:**
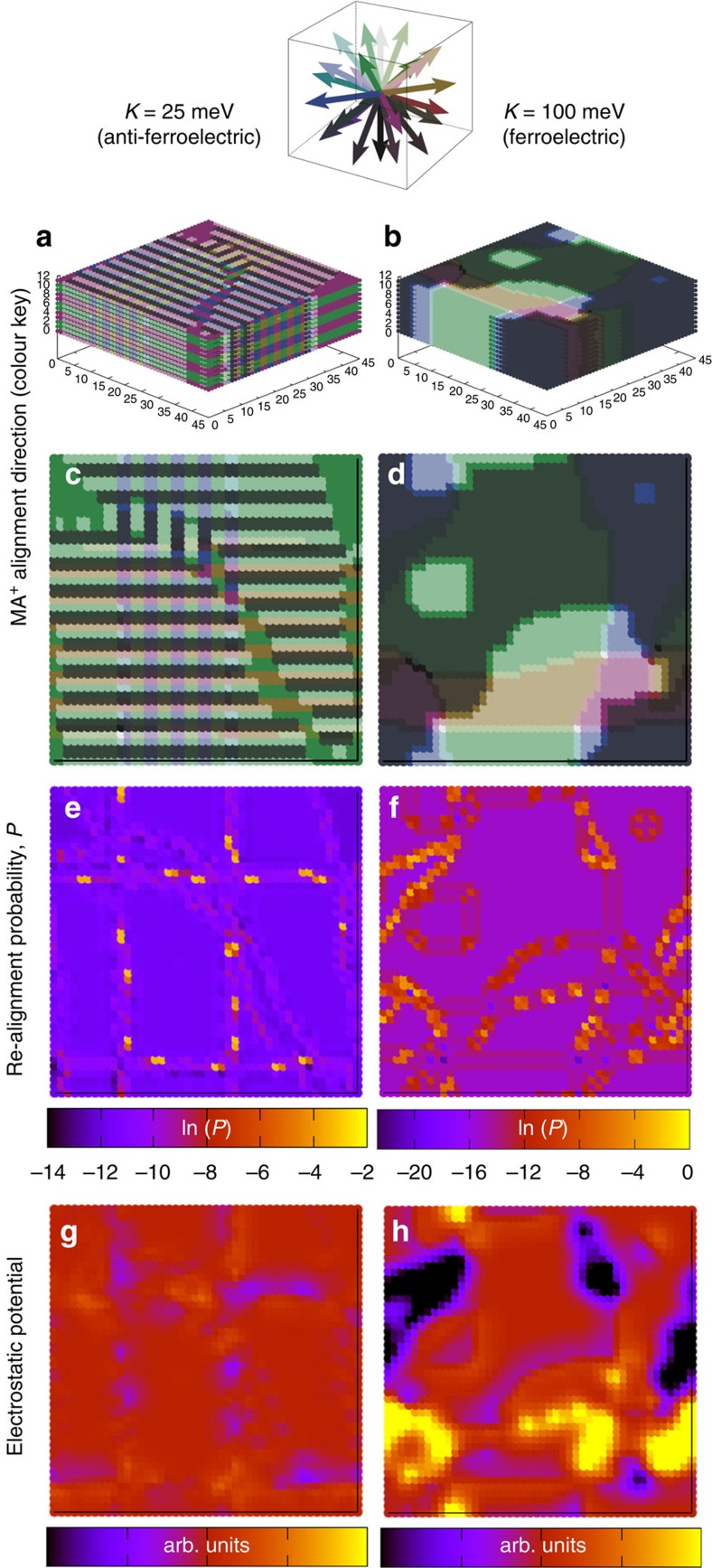
Simulation of interacting MA^+^ dipole alignment on a 3D lattice. Monte Carlo simulation results of thermally activated MA^+^ reorientation in a volume of 45 × 45 × 12 lattice cells (≈28 nm × 28 nm × 7.5 nm) after cooling to 50 K accounting for dipole–dipole interactions and two different cage deformation energies: *K*=25 and 100 meV. (**a**,**b**) MA^+^ orientation for the simulation volume. (**c**,**d**) Single slices taken from **a**,**b**. A vector colour key for the MA^+^ directions in (**a**–**d**) is also shown. (**e**,**f**) The probability that MA^+^ ions at lattice locations corresponding to those shown in **c**,**d** will rotate on their next trial move (**d** shown for 100 K). The scale bar corresponds to the logarithm of the probability of reorientation. (**g**,**h**) Corresponding maps of electrostatic potential resulting from the dipole orientations (arbitrary (arb.) linear units).

**Figure 6:**
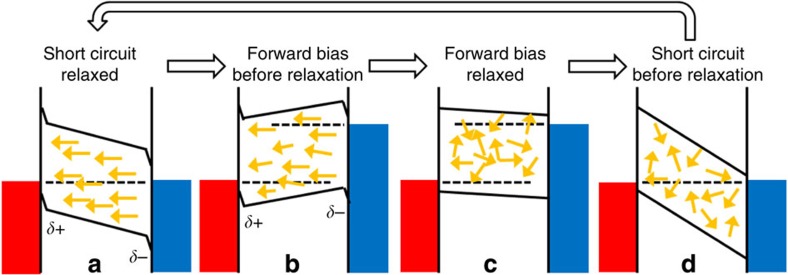
Consequence of polarized domain formation for device behaviour. Schematic energy level diagram indicating the possible consequence of polarized domain formation in the presence of a built-in field on device behaviour. Yellow arrows indicate ferroelectric domain polarization direction, the dotted lines represent the Fermi or quasi-Fermi levels in the device and the solid lines represent the conduction and valence band energies. (**a**) Short circuit, equilibrium conditions. Ferroelectric domains have aligned to screen the built-in potential of the device. This reduces the strength of E-field in the bulk of the film, reducing the collection efficiency of any photo-generated charge carriers. (**b**) Under the application of a forward bias, the internal E-field is reduced or reversed, this allows the thermal randomization of the polarization directions. (**c**) Once the device has had time to relax to the forward bias conditions, there is little or no preferential orientation of ferroelectric domains. (**d**) When the device is returned to short circuit, the built-in potential is not screened by a net polarization direction of ferroelectric domains. This results in a stronger E-field in the bulk of the material and more efficient charge collection. The random domain alignment is gradually lost as the device relaxes back towards the equilibrium condition in **a**.

The experimental ratio of purely elastic-to-elastic plus quasielastic scattering, *R*_*i*_ was determined as a function of *Q* (within the measured range 0.18–1.8 Å^−1^) for each rotational mode *i* according to:


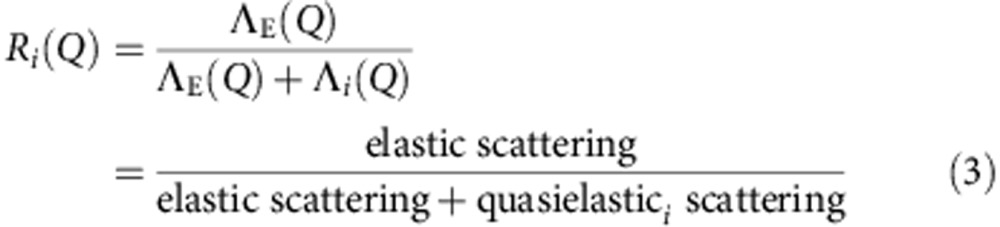


where *Λ*_*E*_(*Q*) is the area under the elastic peak (green curve in the inset in Fig. 3a), and *Λ*_*i*_(*Q*) is the total area under the fitted Lorentzian *i* (purple or orange dotted line in the inset in Fig. 3a). This ratio was fit using the following expression containing the EISF, which accounts for the fraction of scatters not involved in the rotation (1−*x*_*i*_):


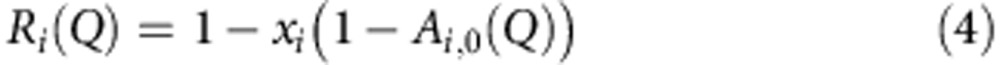


The functional form of the EISF for a given rotational mode, *A*_*i*,0_(*Q*), is entirely specified by the geometry of the molecule's movement; thus, the only free parameters for the fit are the fraction of active scatters, *x*_*i*_, and the type of rotational model itself (different possible forms of *A*_*i*,0_(*Q*) corresponding to different rotational models are given in Supplementary Table 2 and Supplementary Note 2).

### Monte Carlo simulation

The dipole domain simulation extends our previous calculations to three dimensions (which suggested that spontaneous antiferroelectric domains formed for 2D strain-free calculations)^53^. It describes a pseudo-cubic lattice of dipoles (representing the MA^+^ ions with polarization vector **p**_*i*_ for the *i*th dipole), which experience contributions to their energy from interaction with the unshielded applied E-field (**E**_**0**_), dipole–dipole interactions with the near-neighbour dipoles and the contribution due to local cage deformation. The resulting Hamiltonian describing the energy of the system is:


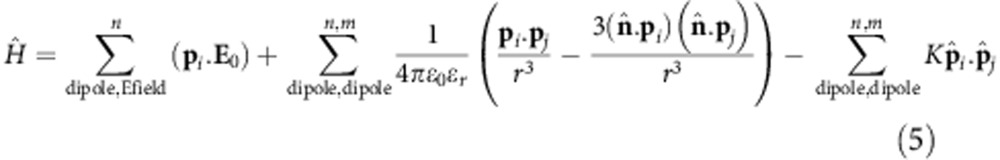


where the unit vector 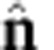 corresponds to the direction between the dipoles and *r* is the distance between them and. *ɛ*_*0*_ is the permittivity of free space. *K* is coupling constant found from density functional theory calculations such that *K*≥0. Our strain value (*K*) comes from a PBESol density functional theory calculation on a 4 × 4 × 4 supercell expansion, where we rotate a single MA dipole while leaving the other 63 aligned. The value we get from this calculation is 150 meV, which we distribute across the nearest six neighbours. However, this is only one measure of this value. Owing to inaccuracies in the electronic structure methods and the model structures, this must be considered to be a first approximation to this value. We are also in the situation where our evaluation of the dipole interaction is as a point dipole, with no dielectric screening. Further, more subtle steric interactions between the molecule and the inorganic cage will result in a very complicated potential energy landscape. As such, we cannot make a firm prediction of which of the different cases (strain=25 meV per face, strain=100 meV per face) is the most realistic condition, and instead perform a sensitivity analysis by looking at the two different regions of the parameter space we observed. The term involving *K* couples alignment or anti-alignment of dipoles to cage deformation, the *ab initio* calculations suggest that this deformation penalizes anti-alignment of nearest neighbour dipoles.

A Metropolis algorithm is used to randomly realign randomly chosen dipoles (*i*) to a new direction. If the energy change of the lattice (Δ*E*) corresponding to the new dipole direction (computed using unshielded dipole–dipole interactions, *ɛ*_r_=1, since the effect of the surrounding lattice on screening is not yet quantified) is negative (exothermic), then the realignment is accepted. If the energy change is positive (endothermic), the dipole realignment is only accepted if *γ*<exp[−Δ*E*/*k*_B_*T*], where *γ* is randomly chosen between 0 and 1, *k*_B_ is Boltzmann's constant and *T* is the temperature. For numerical efficiency, the dipole–dipole interactions were limited to three surrounding lattice units, and, consistent with the QENS results described here and the quantum mechanical MD simulations^53^, dipole directions were limited to alignment along the faces, edges or corners of the lattice cages. The input parameters for the simulation were the polarization of the unshielded MA^+^ ion (calculated to be 2.29 D; ref. 20) and the unit cell spacing (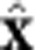, 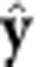 and 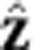 are the unit lattice vectors assumed to be 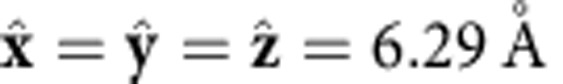, consistent with the neutron diffraction pattern at 298 K; Supplementary Fig. 5). The simulation ignores contributions from MA^+^ ion inertia, which appears reasonable given the observation of discrete hopping events from the QENS analysis. The lattice size simulated was 45 × 45 × 12 cages (corresponding to ∼28 × 28 × 7.5 nm) with periodic boundary conditions. The dipole directions in the lattice were initially randomized at a high temperature (800 K), the simulation was run by attempting to realign the dipoles at random sites according to the criteria described below. The lattice was rapidly cooled in 50 K steps to ensure that the size of any domains formed was less than the simulation volume.

In order to ascertain which dipoles were more likely to rotate for a given configuration, the probability, *P*, that a dipole will reorientate to a new direction on the next Monte Carlo trial was calculated for each dipole within the simulated sample:


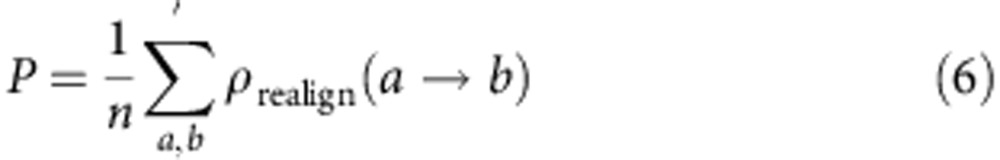


where *a* labels the current polarization state, *b* labels one of the *n*=25 new possible polarization states (not including the current alignment) and *ρ*_realign_(*a*→*b*) is the Monte Carlo probability for a transition from alignment state *a* to *b* given by:


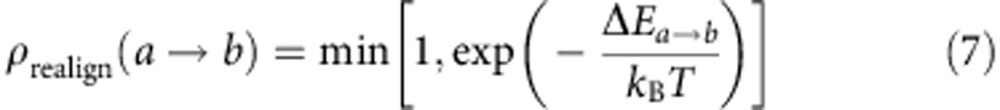


where Δ*E*_*a→b*_ is the energy change of the dipole upon a transition from state *a* to state *b* calculated as the difference in the Hamiltonian (equation 5) between the new and current polarization states.

Approaching thermal equilibrium, the dipole potential felt at a given lattice site (*i*) can be calculated by summing the potential contributions from all other lattice sites (*j*) up to a cut-off radius of five unit cells:


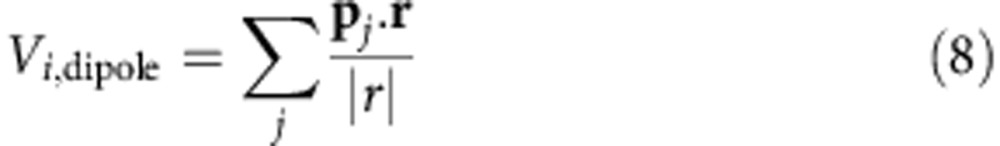


where **r** is the vector between sites *i* and *j*. The potential only explicitly accounts for the polarization field of the MA^+^ ions. Contributions from the surrounding cage are either parallel or antiparallel to the dipole alignments and will thus scale calculated potentials linearly, we have not calibrated this effect so the quoted potentials are in arbitrary units.

## Additional information

**How to cite this article:** Leguy, A. M. A. *et al*. The dynamics of methylammonium ions in hybrid organic–inorganic perovskite solar cells. *Nat. Commun*. 6:7124 doi: 10.1038/ncomms8124 (2015).

## Supplementary Material

Supplementary Information Supplementary Figures 1-26, Supplementary Tables 1-2, Supplementary Notes 1-2 and Supplementary References
