# Supplementary material for: The dynamics of methylammonium ions in hybrid organic–inorganic perovskite solar cells
Source: Nat Commun. 2015 May 29;6:7124. doi: 10.1038/ncomms8124 (PMC4458867; doi:10.1038/ncomms8124)
Supplement: Supplementary Information — Supplementary Figures 1-26, Supplementary Tables 1-2, Supplementary Notes 1-2 and Supplementary References [file ncomms8124-s1.pdf]

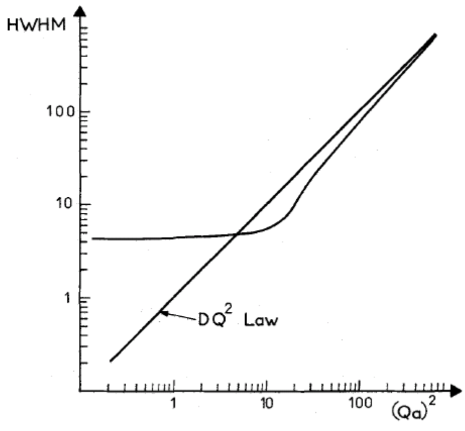

Supplementary Fig. 1. Ruling out constrained diffusion. Reproduced from supplementary reference [1]. Variation of the half width half maximum (HWHM) of the broadened component of the scattering law for diffusion inside a sphere of radius a, expressed in D/a^2 energy units, versus $(Qa)^2$. The HWHM of the DQ^2 law is also represented. These calculations show that confined diffusion within a given potential (e.g. a pore) can also yield a QENS Lorentzian profile that, *at sufficiently low Q*, has a FWHM independent of Q. Under these circumstances, a diffusive motion could be misinterpreted by a rotational motion. In the 1980s, Dianoux and Volino worked on this problem to try to understand and model water (or small molecule) diffusion inside pores, cavities, membranes, etc. Initially they calculated S(q,w) for spherical potentials[2] and then generalised for anisotropic potentials.[3] The graph below if for the predicted HWHH for diffusion inside an impenetrable sphere of radius $r=a$; the other line is simple Fickian diffusion. The key feature is that a kink emerges around $(Qa)^2$ = 16-20 Å^{-2}. We can therefore estimate approximately where a characteristic kink on the FWHM should occur: for a sphere of radius a = 3.15 Å, we should observe a change of slope between Q^2 = 1.6 Å^{-2} and 2.0 Å^{-2}. The flat behaviour observed in **Fig. 3b** (main text) for Q^2 ranging from 0.52Å^{-2} to 3.1Å^{-2} allows ruling out this type of motion. Moreover, the same argument allows ruling out any similar constraint diffusion with a radius larger than 2.6 Å, which given the steric hindrance within the cage and the size of the MA^+ ion is unlikely.

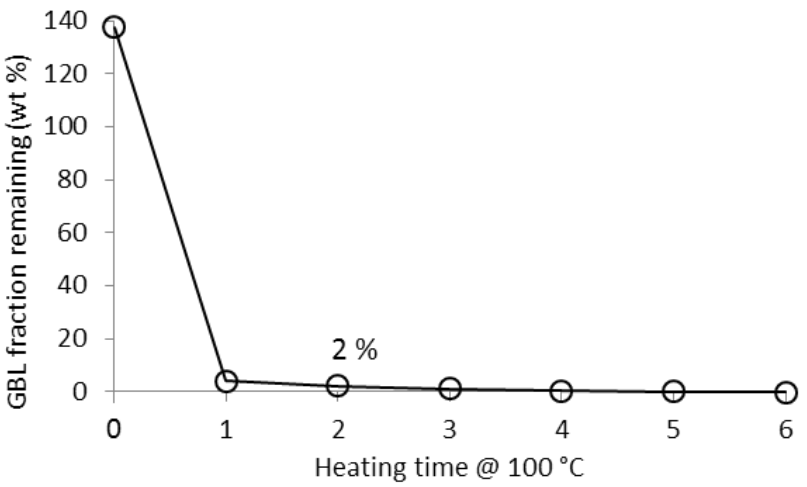

Supplementary Fig. 2. Possible influence of residual GBL in the sample. The percentage by weight of GBL relative to MAPI remaining in the sample material at different heating times starting from the precursor solution PbI_2 + CH_3NH_3I in GBL. The sample of MAPI was prepared as described in the methods section. The mass of the precursor solution was monitored during the annealing step at 100 °C for 6 hours under ambient conditions in a fume hood (the surrounding air was at ~ 20 °C and relative humidity ~ 20 %). The proportion of the sample's total mass that can be attributed to MAPI is shown as a function of time. The results suggest that after 2 hours of annealing the sample used for our experiments may have contained approximately 2 % GBL by weight which would correspond to about 13 % of the hydrogen nuclei in the material (since $M(CH_3NH_3PbI_3)$ = 461.01 g mol^{-1} and $M(C_4H_6O_2)$ = 86.09 g mol^{-1} and both compounds contain an identical number of hydrogens).

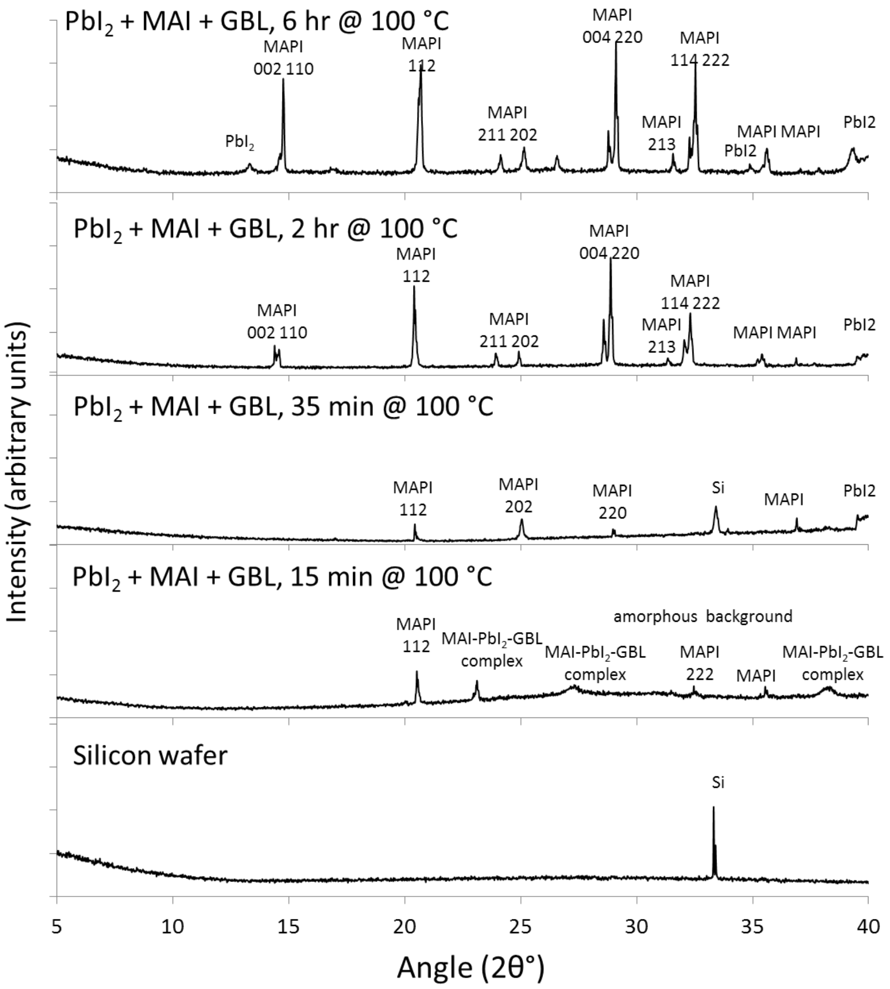

Supplementary Fig. 3. Development of sample crystal structure during preparation. X-ray diffraction patterns of a mixture of PbI_2, CH_3NH_3I and GBL, drop cast on to silicon wafers (prepared as described in the main text and **Supplementary Fig. 2**), and heated at 100 °C, for different times before measurement using an X'pert pro PANalytical diffractometer (Cu K_α, 40 mA, 40 kV, 5° - 40°) at room temperature. The initial thickness of the precursor drop was approximately 2-3 mm with a diameter of approximately 20 mm. The drop thickness was similar to that used in the sample preparation. The measurements indicate that after 15 minutes of heating at 100 °C MAPI crystals had begun form from the solution, the slightly elevated background signal between $2\theta = 20°$ and 40° is likely to correspond to liquid GBL. Also present are broad, unidentified, peaks that we tentatively assign to a MAI-PbI_2-GBL solvated complex which may form as an intermediate prior to the formation of MAPI crystals. After 35 minutes at 100 °C no indication of these peaks remained although traces of the slightly elevated background still remain. After 2 hours of heating the material indicated only the presence of crystalline MAPI with trace PbI_2. Further heating to a total duration of 6 hours followed by leaving the sample at room temperature overnight resulted in almost no change in the XRD pattern: crystalline MAPI with trace lead iodide. The x-ray diffraction patterns suggest that with the exception of traces of PbI_2 (which is frequently observed in XRD patterns of MAPI thin films for devices and does not contribute significantly to the incoherent neutron scattering intensity) no other crystalline phases were present in the material.

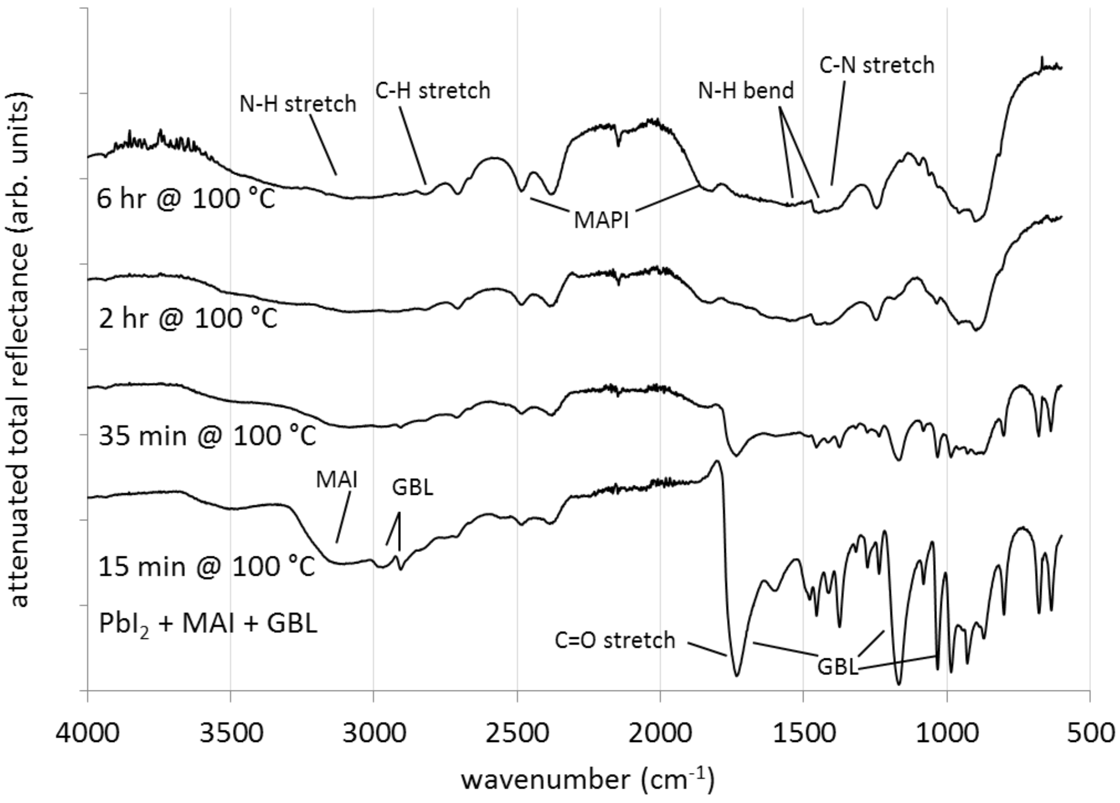

Supplementary Fig. 4. Monitoring the GBL FTIR signature during sample preparation. Fourier transform infrared spectra measured using attenuated total reflectance on the materials shown in **Supplementary Fig. 3**. These were was scraped onto a Specac diamond sample stage mounted on a Perkin Elmer Spectrum 100 FTIR spectrometer at room temperature. The powdered material was not mixed with KBr to reduce the possibility of solvent loss and Br^- - I^- ion exchange[4] in the samples prior to measurement. The spectrum collected after 15 minutes at 100 °C indicates a strong signature of GBL characterised by a number of strong absorption peaks between 600 – 1700 cm^{-1} and weaker peaks at 2900 – 3000 cm^{-1}, a particularly strong absorption band is the C=O stretch at approximately 1700 cm^{-1}.[5] After 35 minutes of heating the signature of GBL is significantly reduced, and after 2 hours the C=O peak has disappeared, suggesting little if any GBL remains in the material. The spectral features after 2 and 6 hours of heating appear broadly consistent with the FTIR spectrum for MAPI pressed into KBr pellets shown in reference[6] and the features seen for FTIR spectra of thin films in reference.[7]

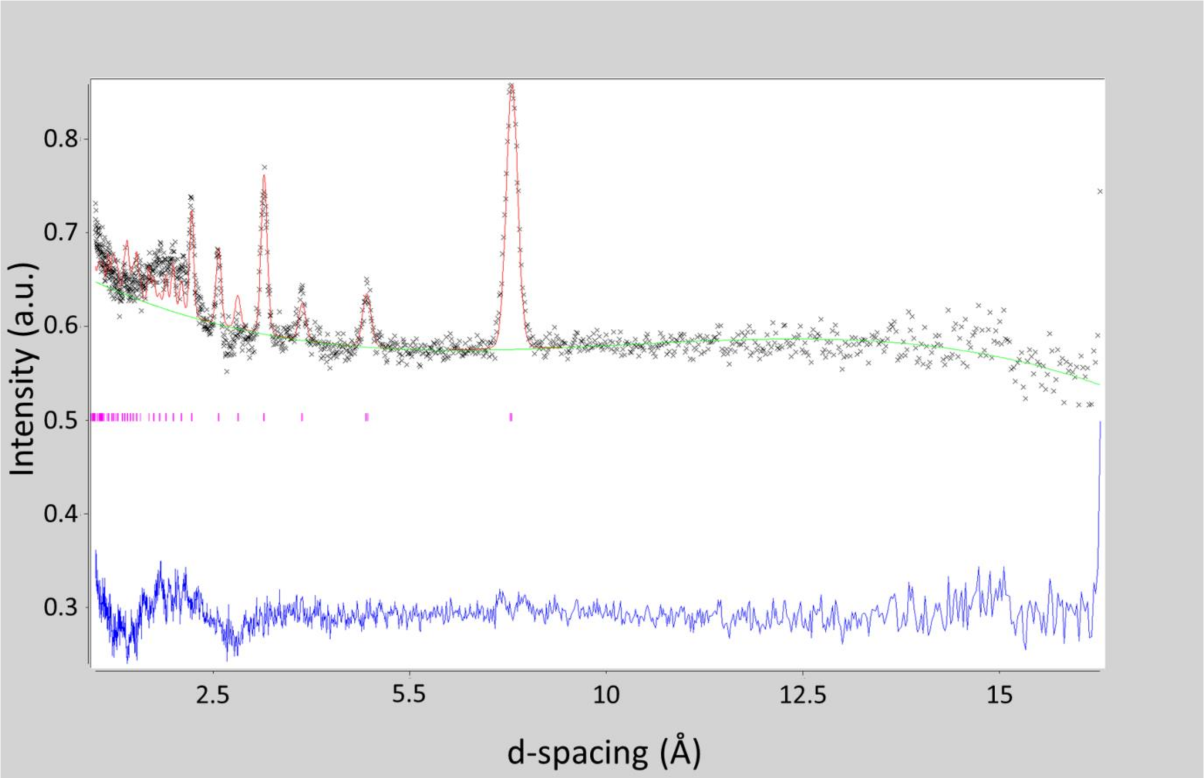

Supplementary Fig. 5. MAPI diffraction spectrum from the GEM spectrometer. Neutron diffraction spectrum of MAPI prepared as described in the methods section. Data collected by GEM using detector bank 2 are represented by black crosses.[8] The green line is the fitted background (fourth order Chebyschev polynomial function) while the red curve shows the expected peak shape from quantum chemical calculations of the relaxed structure. The pink markers indicate the expected position of the diffraction peaks and the blue line shows the residuals between experimental and theoretical data.

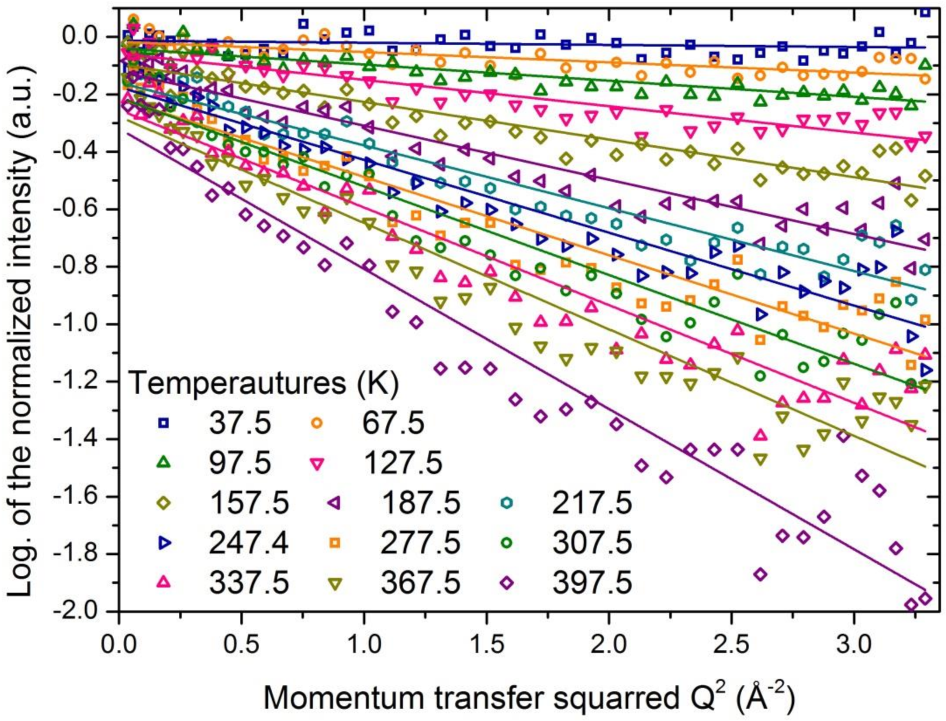

Supplementary Fig. 6. Extrapolation of the 'effective' Debye-Waller factor. The logarithm of the integrated elastic scattering intensity (i.e. for $|\Delta E| \leq 25.4$ μeV) is plotted against Q^2 and linearly fit to extrapolate the effective Debye-Waller factor. As a crude initial approach to explore the scattering data we analysed the scattering data in terms of an 'effective' Debye-Waller factor only as a means to assess the characteristic magnitude of atomic motion in the samples. This does not attempt to assign the broadening of the scattered energy spectra to the rotational modes as presented in the main text, but gives an indication of the extent of atomic motion in the sample under given conditions. At sufficiently low temperatures, below activation of diffusive motion, the mean square displacement, $\langle u^2 \rangle$, can be rigorously interpreted as arising from (T-dependent) proton delocalisation. The incoherent dynamic structure factor was estimated by extrapolating temperature dependent data to T = 0 K to give $S(Q,\omega,T = 0\text{ K})$, which we subsequently used to normalize $S(Q,\omega)$. In the absence of quasielastic broadening, the decrease of the elastic intensity is described by the Debye–Waller factor which corresponds to the mean squared vibrational displacement of the hydrogen atoms from their equilibrium location. Thus the slope of the logarithm of $S(Q,\omega,T)/S(Q,\omega,T = 0\text{ K})$ is proportional to the apparent mean squared amplitude of vibrations, which enables us to infer the value of the Debye-Waller factor (see **Supplementary Fig. 7**).

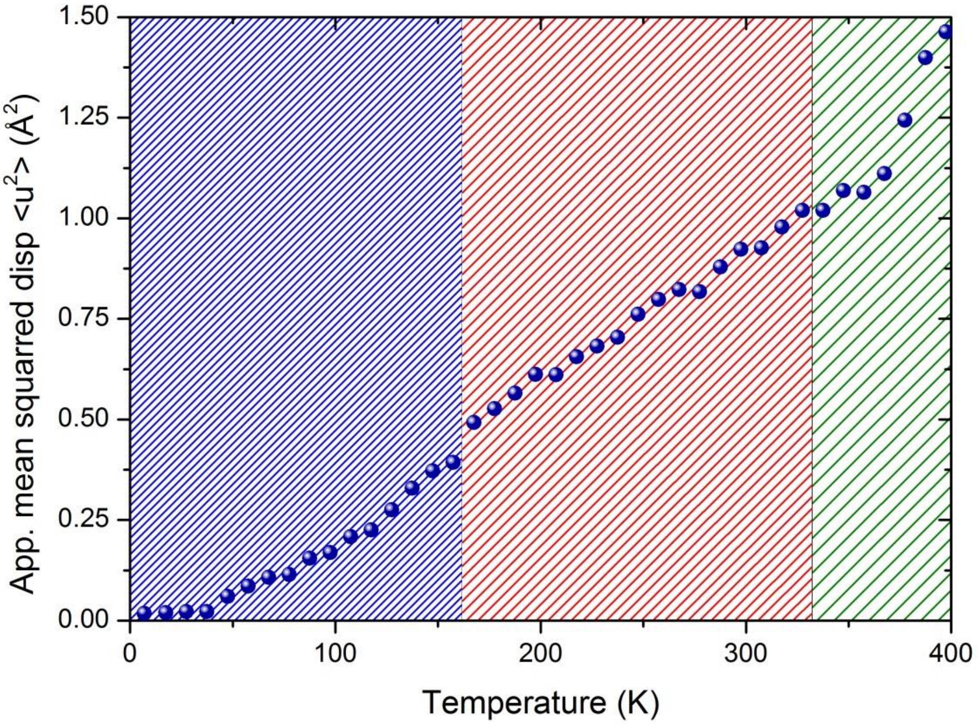

Supplementary Fig. 7. Effective mean squared amplitude of the proton displacement. The <u^2> values are inferred from the fits in **Supplementary Fig. 6** assuming all motion can be described by the Debye-Waller factor, they are of plotted against temperature. The superimposed coloured zones give the limits of the different crystalline phases: orthorobic (blue), tetragonal (red) and cubic (green). The slope of <u^2> with temperature changes significantly at 50 K and around 375 K. These changes are likely to correspond to the onset of new translational or rotational modes. Relatively little change in slope is observed across the orthorhombic-tetragonal crystalline phase transition at about 160 K. No high statistics measurements were collected above 370 K to analyse any potential new mode. We note the freezing point of pure GBL is around 230 K although in the presence of another phase this would be depressed. If significant quantity of liquid GBL were present in the sample and contributing the QENS peak broadening we would expect to see a change in slope of the effective Debye-Waller factor at the melting point, no clear change in slope is observed at or below the pure solvent freezing point attributable to its presence.

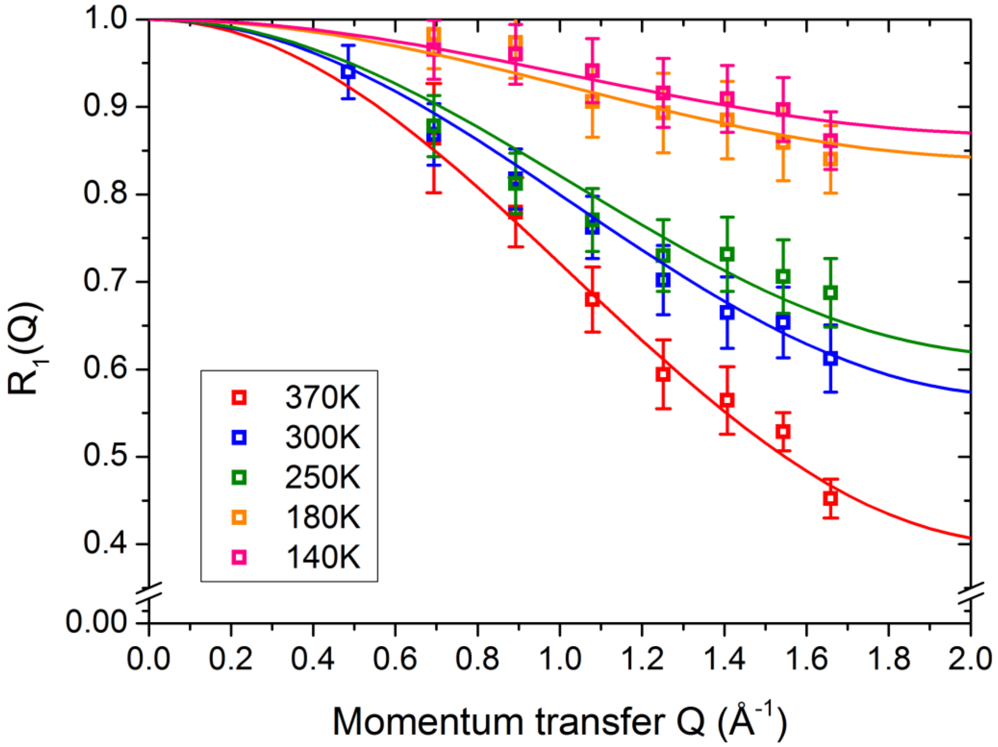

Supplementary Fig. 8. Methyl and/or ammonium rotation around the C-N axis. The elastic to elastic and quasielastic scattering intensity ratio, $R_1(Q)$, of the rotational motion attributed to CH_3 and/or NH_3 about the C-N axis (motion '1' in **Fig. 1** corresponding to the broad Lorentzian in **Fig. 3** main text) is plotted against Q. The solid lines correspond to the fitted EISF function, Equation 4 (main text), using the first line of **Supplementary Table 1** for $a_0(Q)$, and the fitting parameter x_1 for the temperature dependent active fractions. The path geometry defined by $a_0(Q)$ in the fit corresponds to the threefold 'clicking' jump rotation of methyl or ammonium groups.[9,10] There is a negligible difference between the fits using the NH_3 and CH_3 radii thus it is not possible to distinguish between the two possible modes. All the data is well fit by the model. The active fractions, x_1, are presented in **Fig. 3d** (main text).

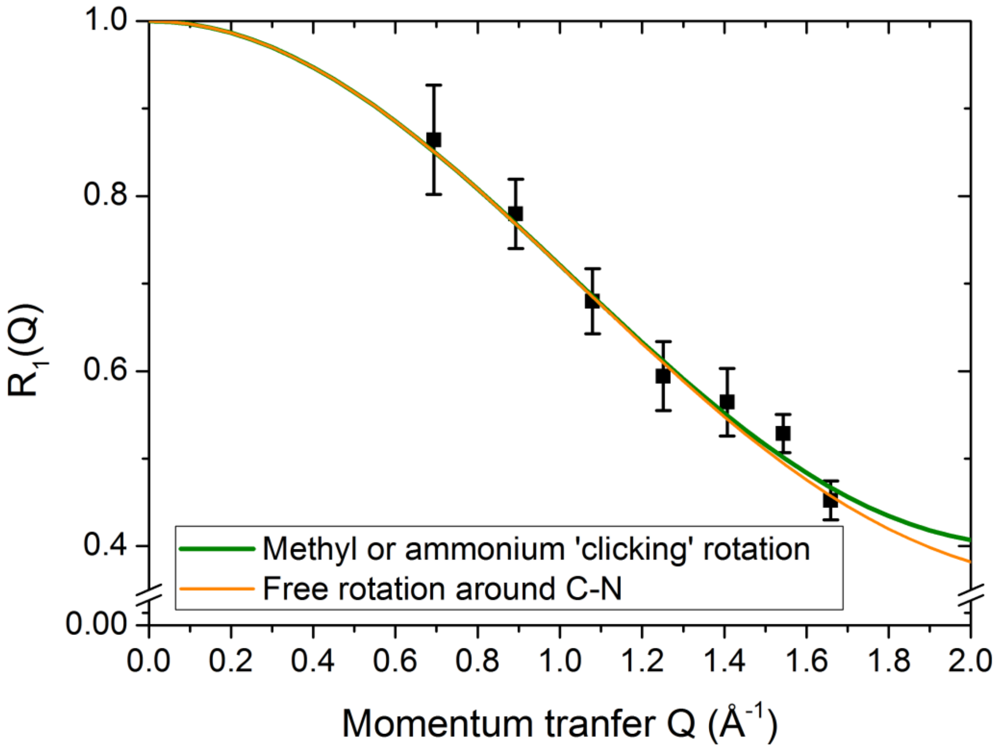

Supplementary Fig. 9. Free rotation or 3-fold 'clicking' of NH_3 and/or CH_3 around the C-N axis? Elastic to elastic and quasielastic scattering intensity ratio, $R_1(Q)$, at 370K corresponding to rotational mode '1' in **Fig. 1b** assigned to the broad Lorentzian in **Fig. 3** in the main text plotted against Q. Fits are shown using EISFs functions described in the first two rows of **Supplementary Table 1** for the N-sites reorientation on a circle of radius 1.033Å for the clicking methyl or ammonium rotation (N = 3) and the free rotation about C-N (approximated by N = 6 since there is no analytical expression for the free rotation on a circle). The square markers represent the elastic to quasielastic scattering ratio. The active fraction at this temperature is 64%. For the momentum transfer range under investigation in this work, it was not possible to distinguish between three sight 'clicking' reorientation and the free rotation around C-N.

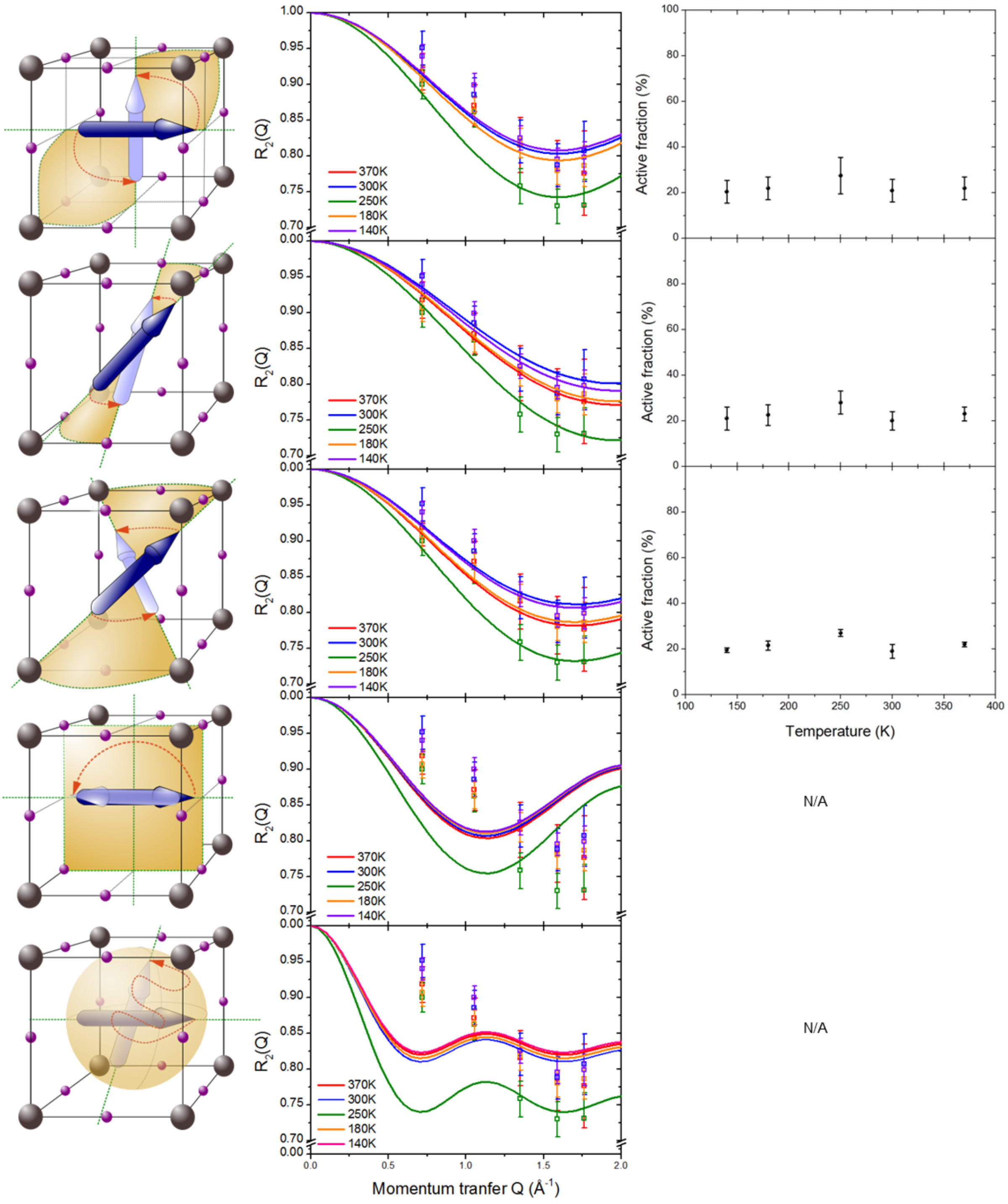

Supplementary Fig. 10. Assessment of different C-N reorientation models at all temperatures. Left-hand column: geometries of the motions of C-N reorientation under consideration. Middle column: comparison between fits of Equation 4 (main text) with corresponding EISF models (given in **Supplementary Table 1**) to the measured ratio of elastic to elastic and quasielastic scattering intensity, $R_2(Q)$. Right-hand column: value of the active fraction, x_2, giving the best fits (if applicable) as a function of temperature. Note that there is possibly a shoulder in the $R_2(Q)$ data at 1.1 Å^{-1}, this could be consistent with a contribution of up to 5% of the mobile MA^+ ions being involved in free rotation or a slight contribution from coherent scattering (see **Supplementary Fig. 13**).

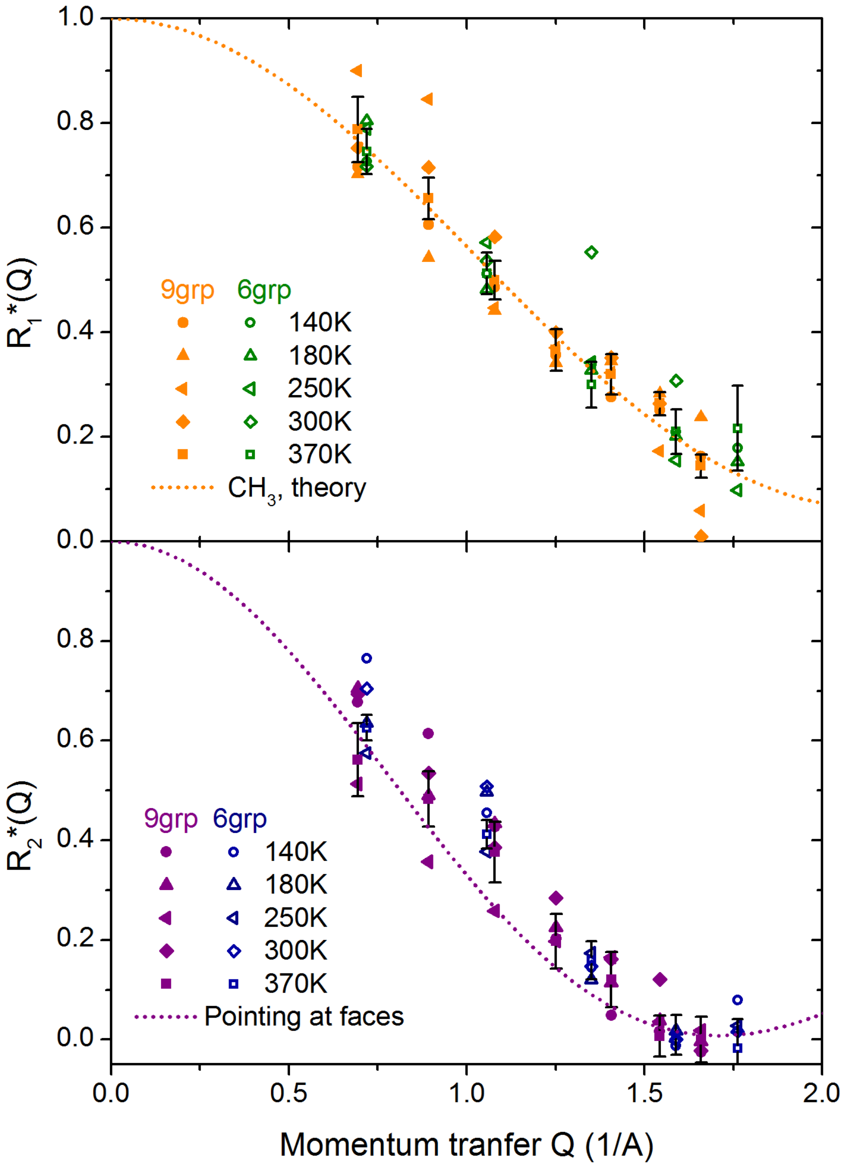

Supplementary Fig. 11. The invariance of $R_i(Q)$ to different Q-grouping and temperatures. To allow easier comparison of the scattering data corresponding to only the active scattering fraction, x_i, we define a corrected elastic to elastic and quasielastic ratio, $R_i^*(Q) = [R_i(Q) - (1 - x_i)]/x_i$. The corrected ratio, $R_i^*(Q)$, is plotted against momentum transfer for different temperatures, and for different numbers of detectors in a group. Upper panel: mode '1', lower panel: mode '2' in **Fig. 1** (main text). The shape of the elastic to quasielastic ratio against momentum transfer Q is expected to be independent of temperature and robust to changes of detector Q groupings which are used in order to improve statistics, at the expense of Q-resolution. The latter is particularly important if the coherent structure factor is significant or crystallinity is present (see **Supplementary Fig. 13**). The validity of the models may thus be checked by evaluating the temperature and Q-grouping dependence of the elastic to quasielastic ratio. If its shape varies significantly with

temperature then the contribution of an additional mode or coherent reflection is likely to have been omitted from the analysis. No significant variation is observed between temperatures or using 6 and 9 detector groups within measurement and fitting uncertainty.

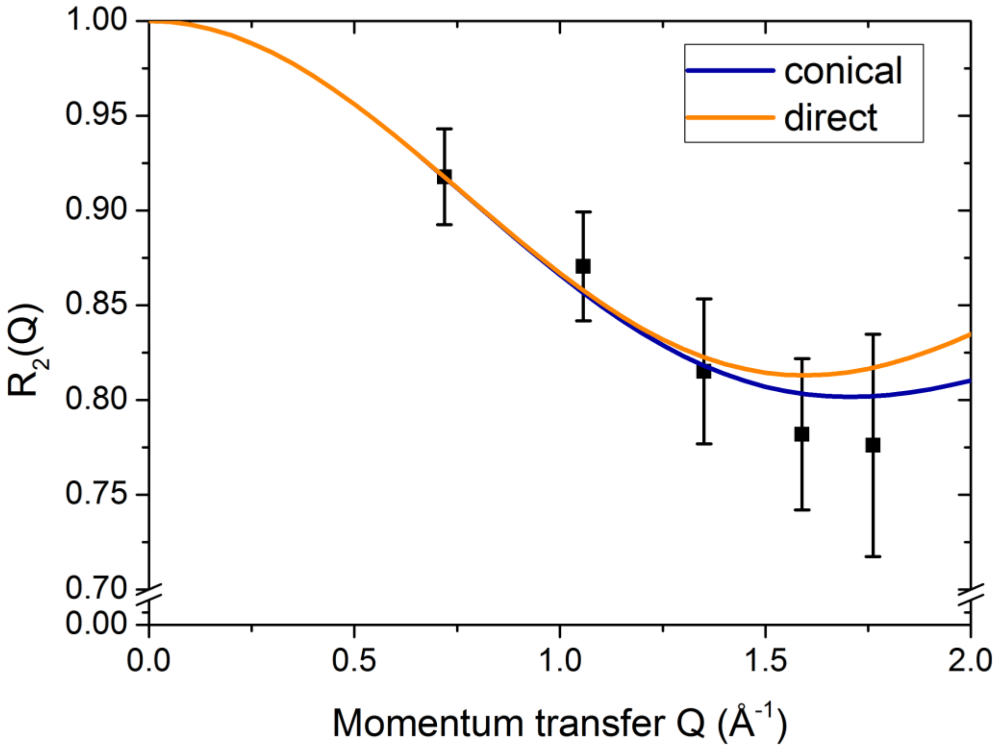

Supplementary Fig. 12. Geometrical path dependence of the EISF. Comparison of the models for two different reorientation paths of the C-N axis between adjacent lattice faces. One path corresponds to "direct" (in orange) and "conical" (in blue) reorientation in the 'face to face' hopping case. Experimental points of the elastic to elastic and quasielastic scattering ratio, $R_2(Q)$, at 370 K are represented by black square markers. The geometry of the motion determines shape of the EISF ratio (**Supplementary Table 1**). The position of the minimum of the theoretical EISF varies depending whether the path between hops is conical or straight. A straight path would correspond to 90° jumps of radius 1.39 Å whereas a conical reorientation between two sites is described by 120° jumps of same radius. Similar reasoning applies for the other jumps under consideration. We found that the conical movement gave better fits in all cases.

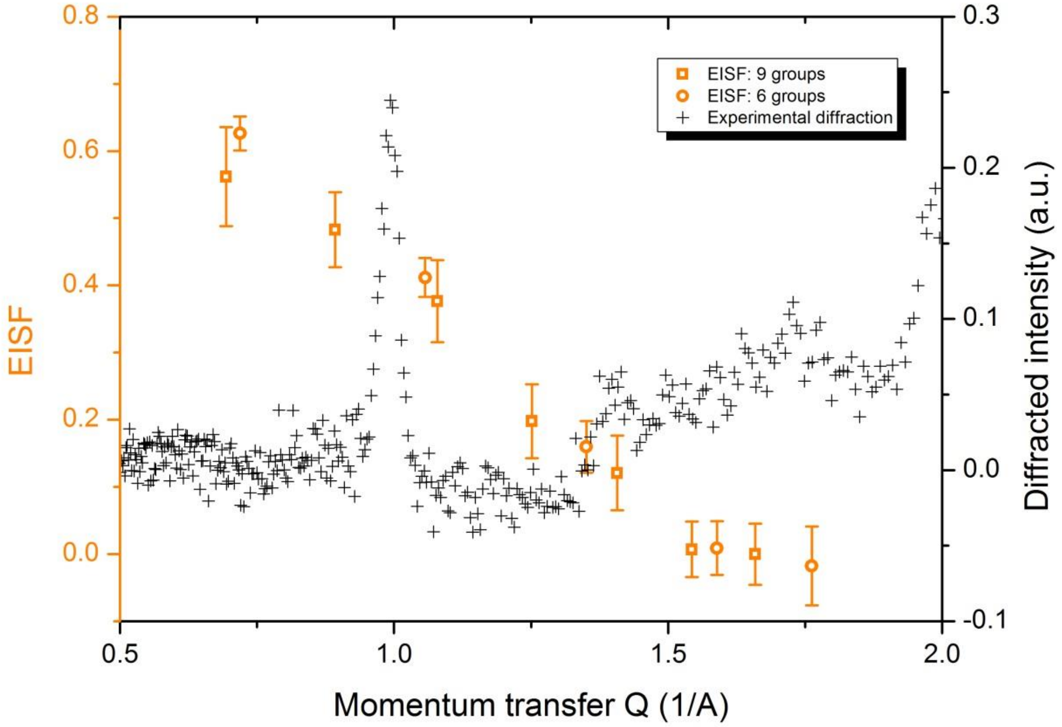

Supplementary Fig. 13. The influence of diffraction on the scattering signal. Influence of coherent signal on the EISF. Comparison between the EISF given by $R_2^*(Q)$ from the elastic to elastic and quasielastic scattering ratio (corrected as in **Supplementary Fig. 11**) at 370K plotted against Q for different detector groupings and the position of diffraction peaks from the spectrometer GEM at ISIS.[8] Removing the detectors possibly affected by coherent neutron diffraction peaks had no significant influence on the shape of the EISF. This confirms that the analysis has not been significantly influenced by diffraction artefacts.

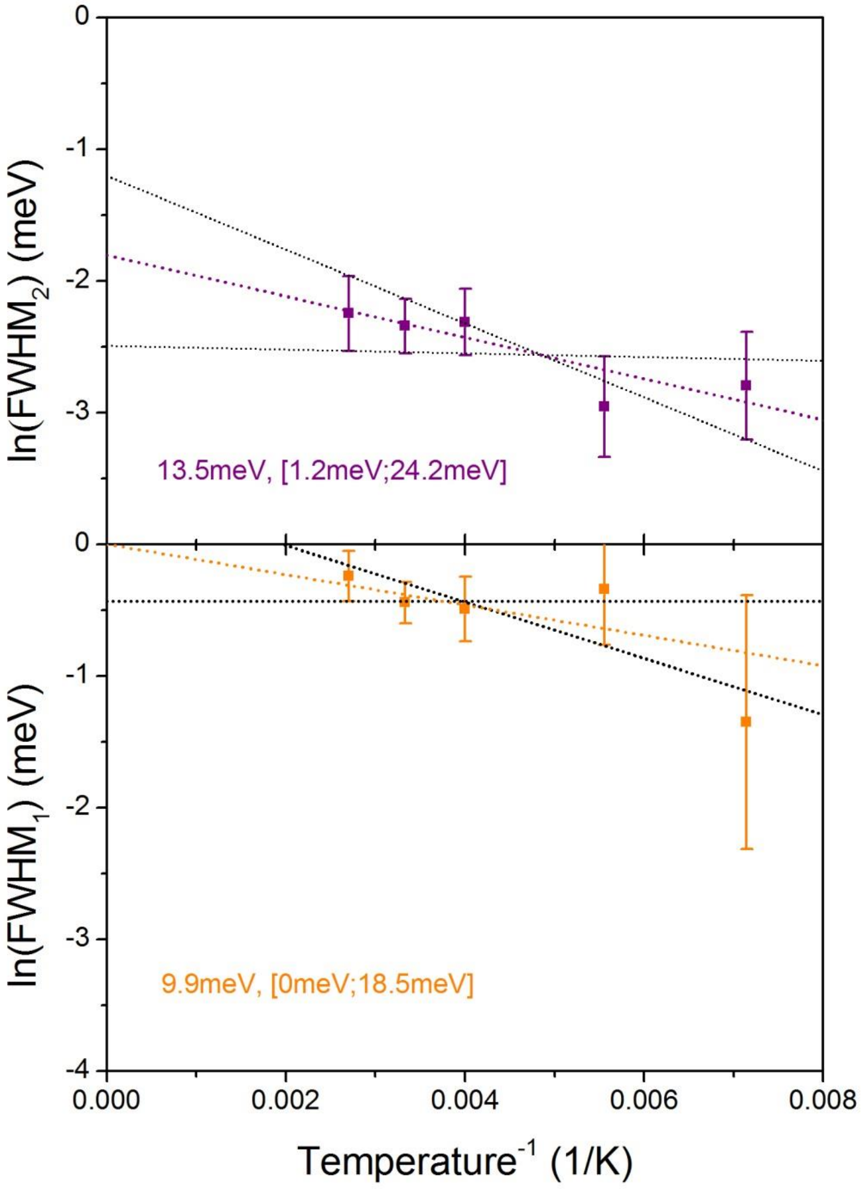

Supplementary Fig. 14. Activation energies for the two rotational modes. The average value of the Lorentzian peak broadening for all *Q*s was extracted at each temperature. Since these rotational processes should be thermally activated, Arrhenius enable the activation energy of each mode to be estimated within measurement uncertainty.Upper panel: rotational mode 1, corresponding to the broader Lorentzian in **Fig. 3**. Lower panel: reorientation mode 2 corresponding to the narrower Lorentzian in **Fig. 3** main text. On each plot, the coloured dashed line corresponds to the best linear fit while the dotted lines stand for the smallest and biggest slopes obtainable within the error bars (the extremal slopes are given on the graph in the form of intervals).

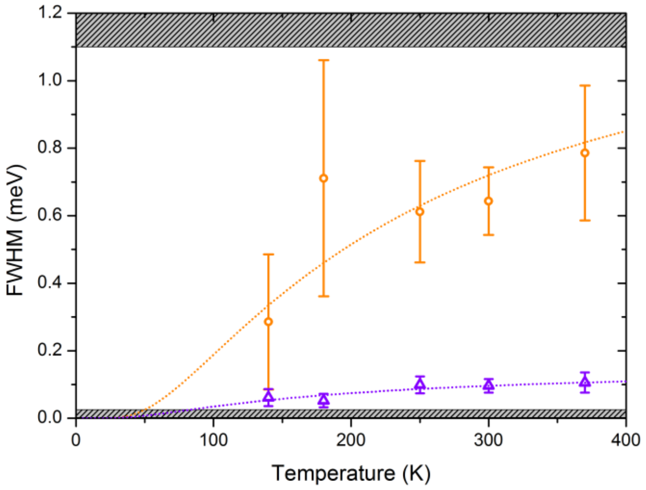

Supplementary Fig. 15. Experimental energy window of the spectrometers. The FWHM for each reorientation (mode 1: orange and mode 2: purple) are plotted on graph together with the limits of the spectrometer. The dotted lines correspond to the temperature dependence of the FWHMs extracted from the Arrhenius plots in **Supplementary Fig. 14**. To interpret the active fractions of scatters presented in **Fig. 4d** (main text), the values of the FWHM were checked against the experimental energy window. The aim of this analysis is to determine when a given motion is expected to enter the measurable energy window, and when it will leave it. Any Lorenzian with a FWHM broader than the measurement interval: [-0.55 eV; 0.55 eV] cannot be fitted unequivocally and will be detected as background (corresponding to the upper limit on the plot). Conversely, any Lorenzian with a FWHM smaller than the instrument resolution FWHM will disappear within the elastic peak (indicated on the graph by the lower limit). Therefore, in the case of the broad Lorenzian (corresponding to the methyl-rotation), the mode is expected to enter the energy-window around 50K, and will not leave it before 400K. For the sharp motion, the FWHM is distinguishable from the resolution above approximately 75K, and is not expected to disappear in the background at any of the investigated temperatures.

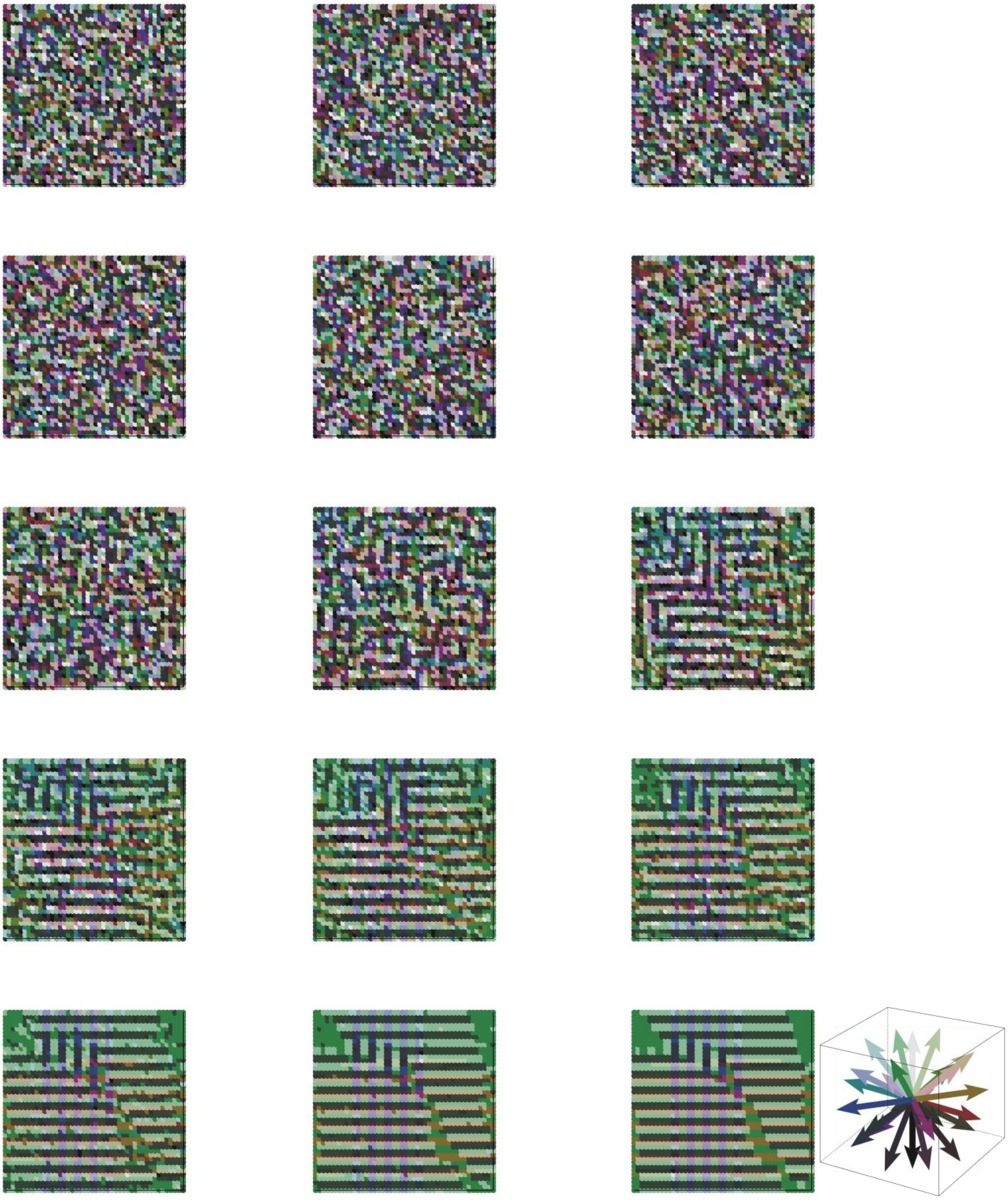

Supplementary Fig. 16. MA^+ dipole orientation during cooling: antiferroelectric case. Slices of the simulated dipole configurations from the K = 25 meV case (dipole direction coloured arbitrarily). Simulation lattice size 45 × 45 × 12. Ran for an average of 500 Monte Carlo steps per site at each temperature, initially randomised at T = 800 K and then cooled down in steps of 50 K. Plots from T = 750 K (top left) to T = 50 K (bottom right) are shown. A colour key for the MA^+ alignment is also shown.

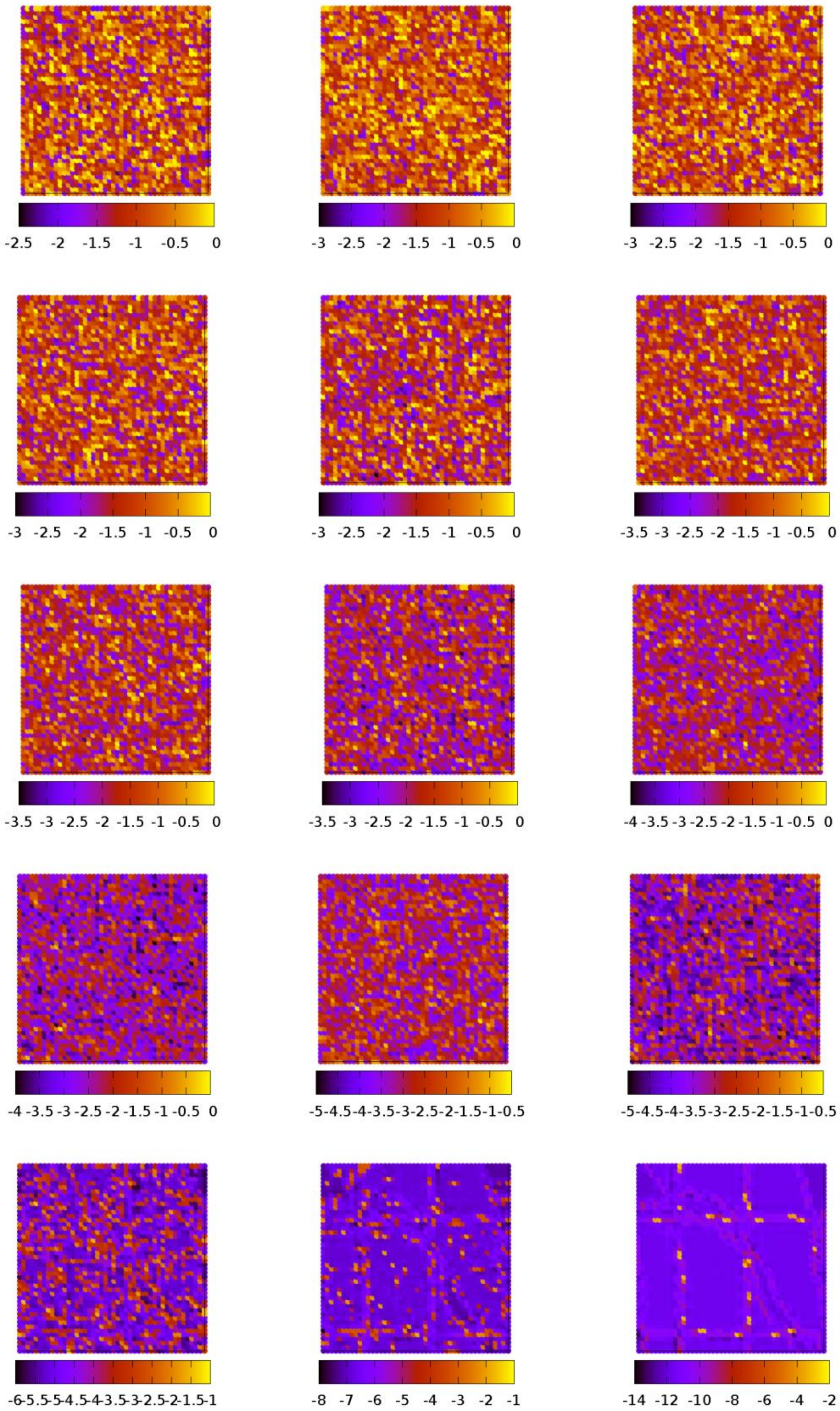

Supplementary Fig. 17. MA^+ rotational probability during cooling: antiferroelectric case. Slices of the reorientation probability, ln(P), for the K = 25 meV case, corresponding to the slices shown in **Supplementary Fig. 16** with temperatures ranging from 750 K (top left) to 50 K (bottom right).

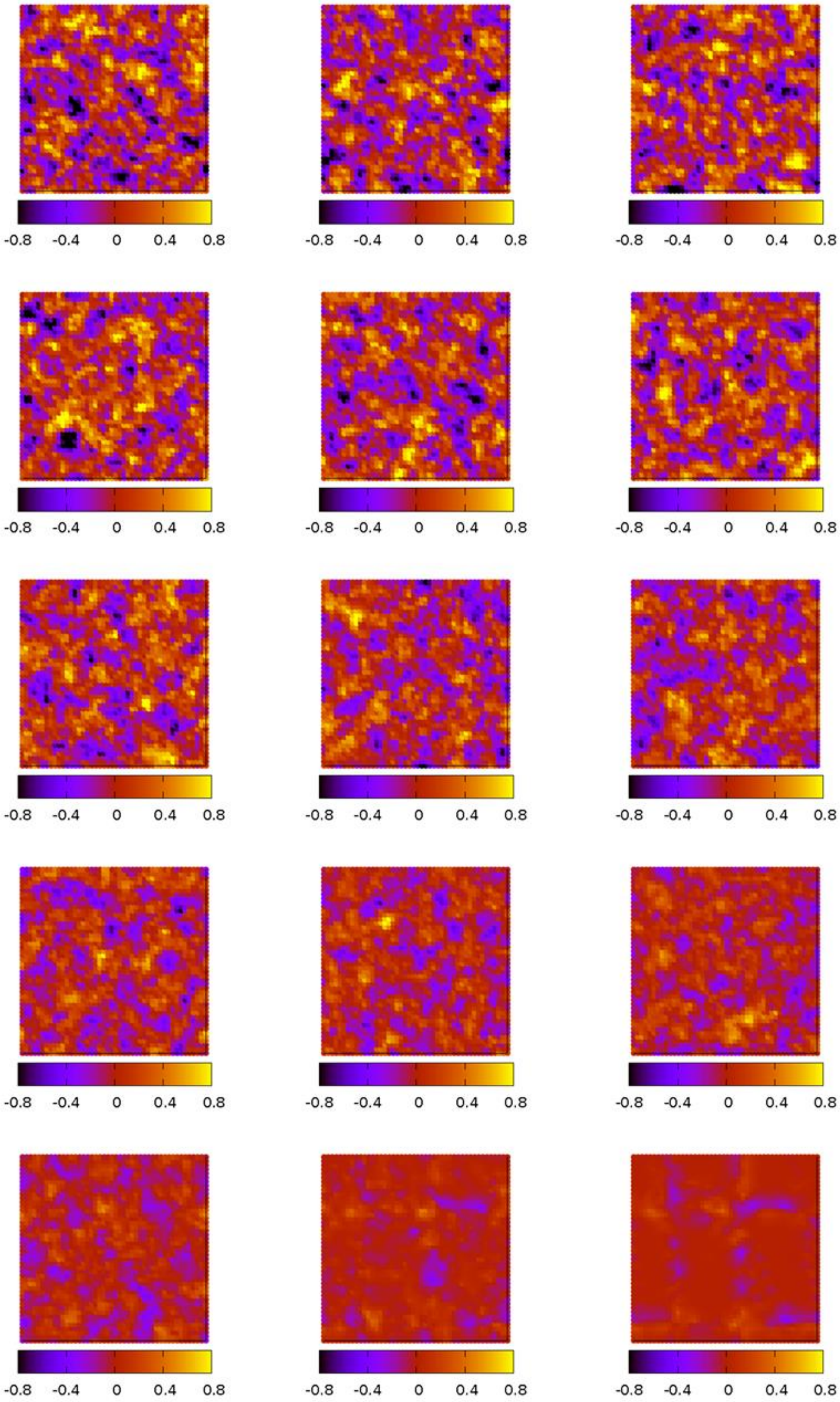

Supplementary Fig. 18. Electrostatic potential during cooling: antiferroelectric case. Slices of the electrostatic potential (arbitrary units) for the K = 25 meV case, corresponding to the slices shown in **Supplementary Fig. 16** with temperatures ranging from 750 K (top left) to 50 K (bottom right).

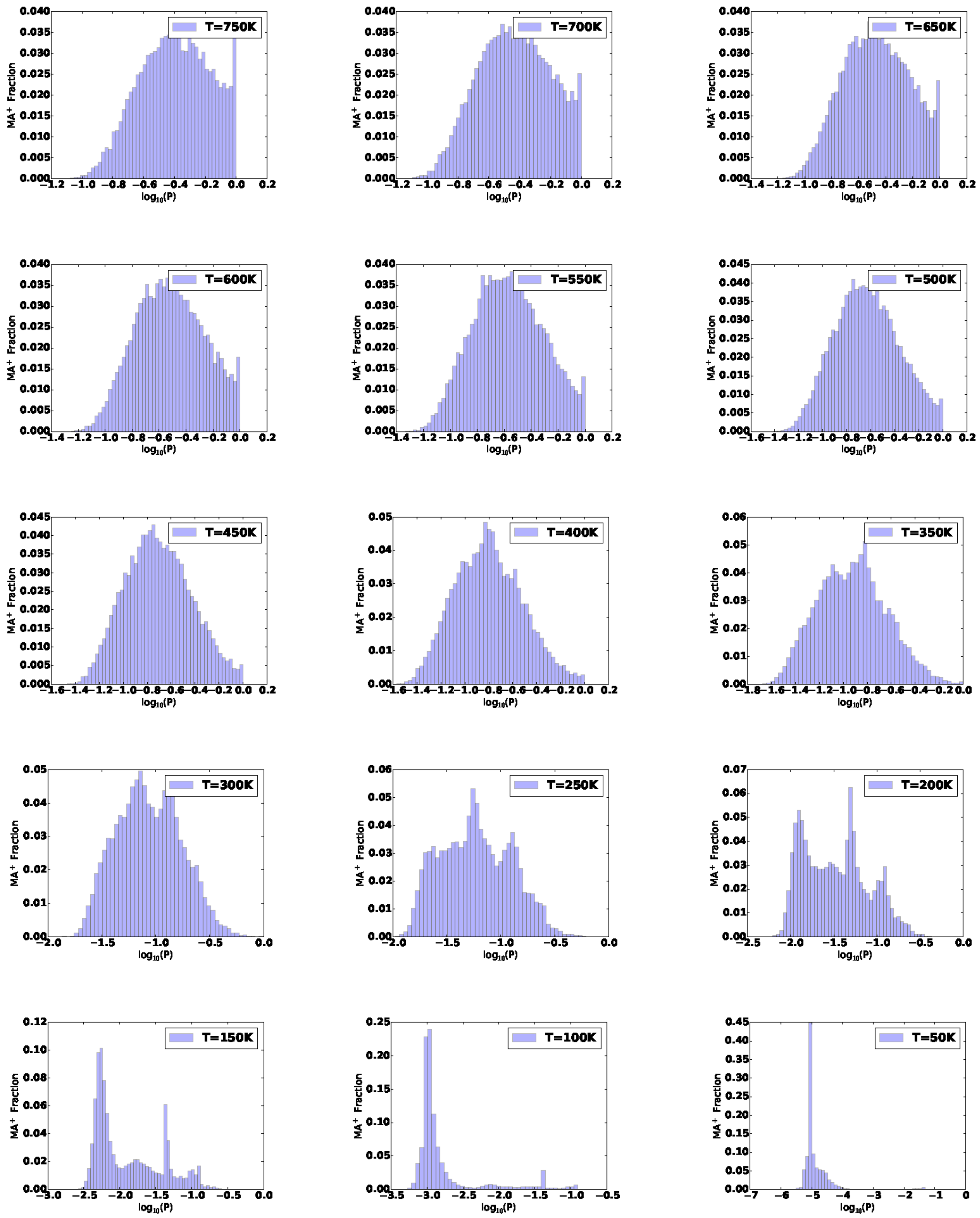

Supplementary Fig. 19. MA^+ reorientation probability distribution during cooling: antiferroelectric case. Histograms from the full simulation lattice described in **Supplementary Fig. 16** for K = 25 meV showing the probability that MA^+ ions will undergo realignment on their next move trial. The temperatures indicated range from T = 750 K (top left) to T = 50 K (bottom right).

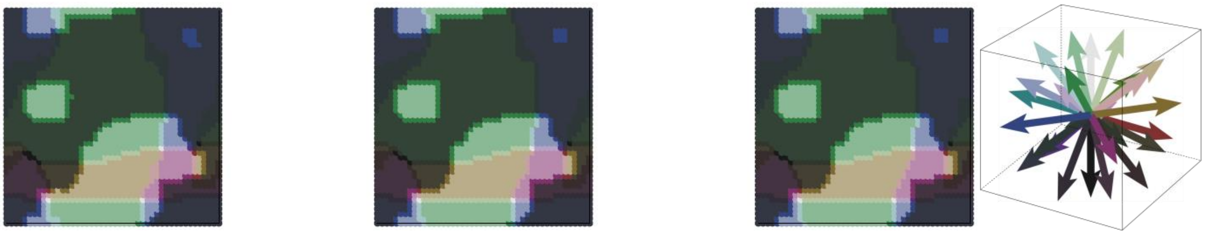

Supplementary Fig. 20. MA^+ dipole orientation during cooling: ferroelectric case. Slices of the simulated dipole configurations from the K = 100 meV case (dipole direction coloured arbitrarily). Simulation lattice size 45 × 45 × 12. Ran for an average of 500 Monte Carlo steps per site at each temperature, initially randomised at T = 800 K and then cooled down in steps of 50 K. Plots from T = 750 K (top left) to T = 50 K (bottom right) are shown. A colour key for the MA^+ alignment is also shown.

right).

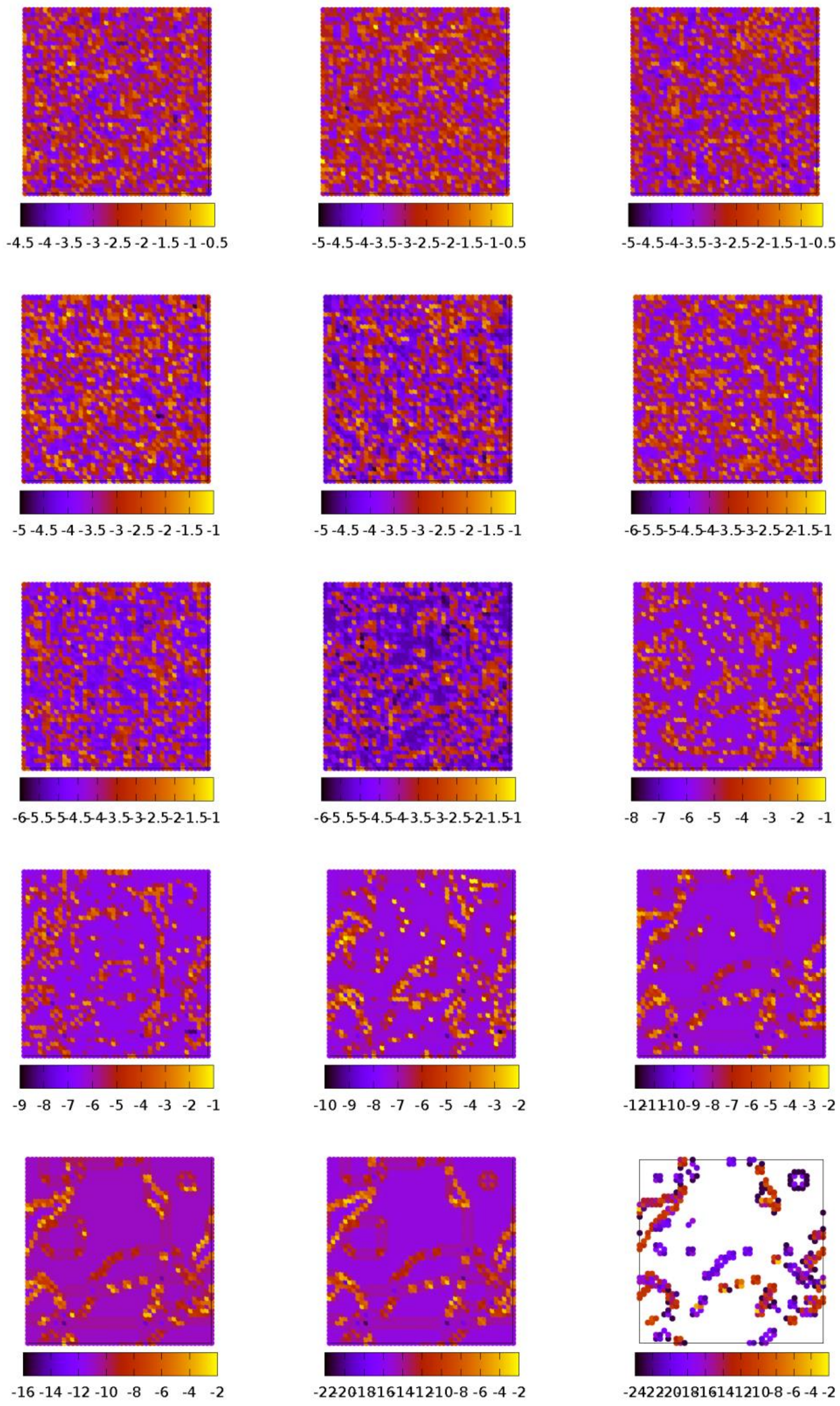

Supplementary Fig. 21. MA^+ rotational probability during cooling: antiferroelectric case. Slices of the reorientation probability (logarithmic scale) for the *K* = 100 meV case, corresponding to the slices shown in **Supplementary Fig. 20** with temperatures ranging from 750 K (top left) to 50 K (bottom right).

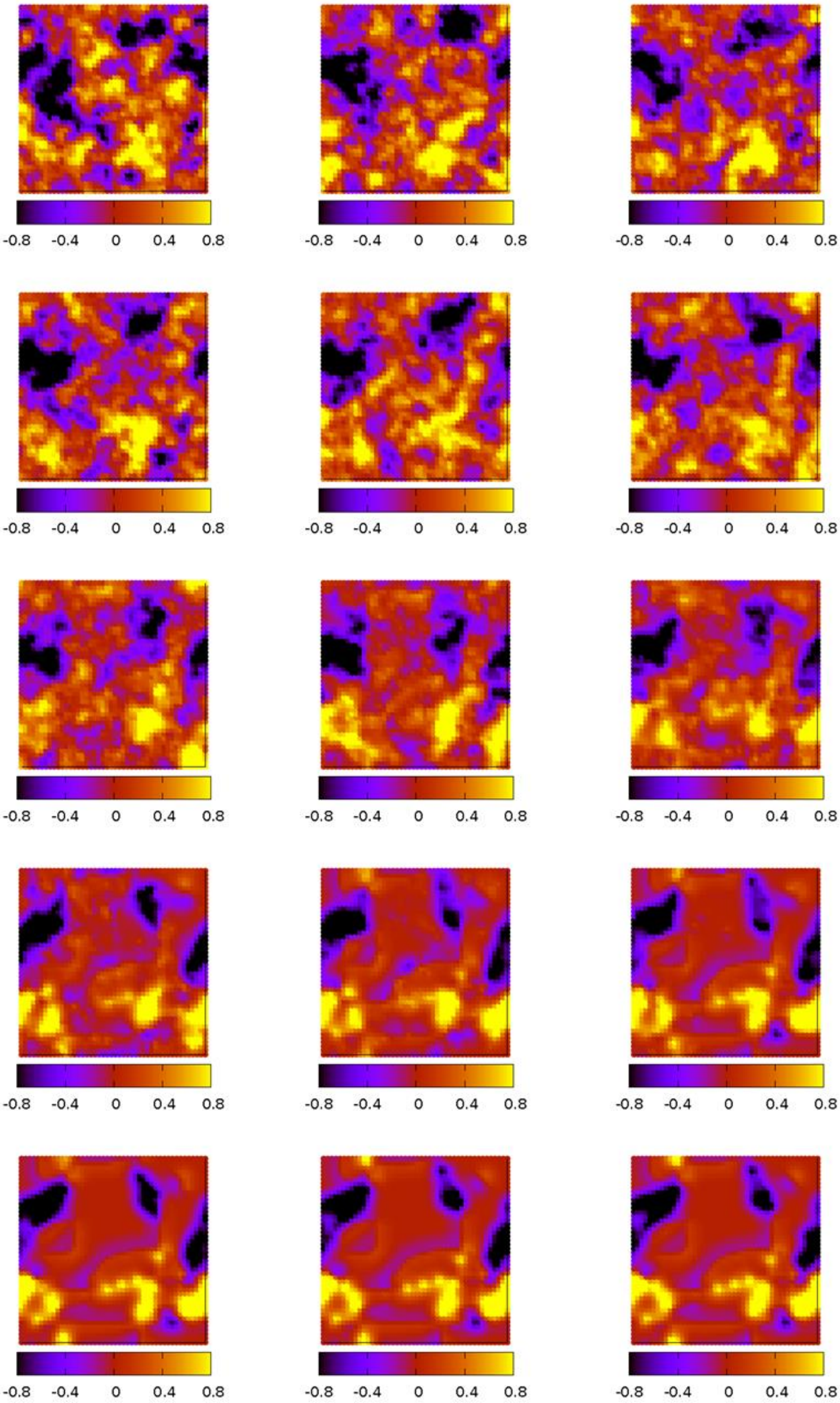

Supplementary Fig. 22. Electrostatic potential during cooling: ferroelectric case. Slices of the electrostatic potential for the *K* = 100 meV case (arbitrary units), corresponding to the slices shown in **Supplementary Fig. 17** with temperatures ranging from 750 K (top left) to 50 K (bottom right).

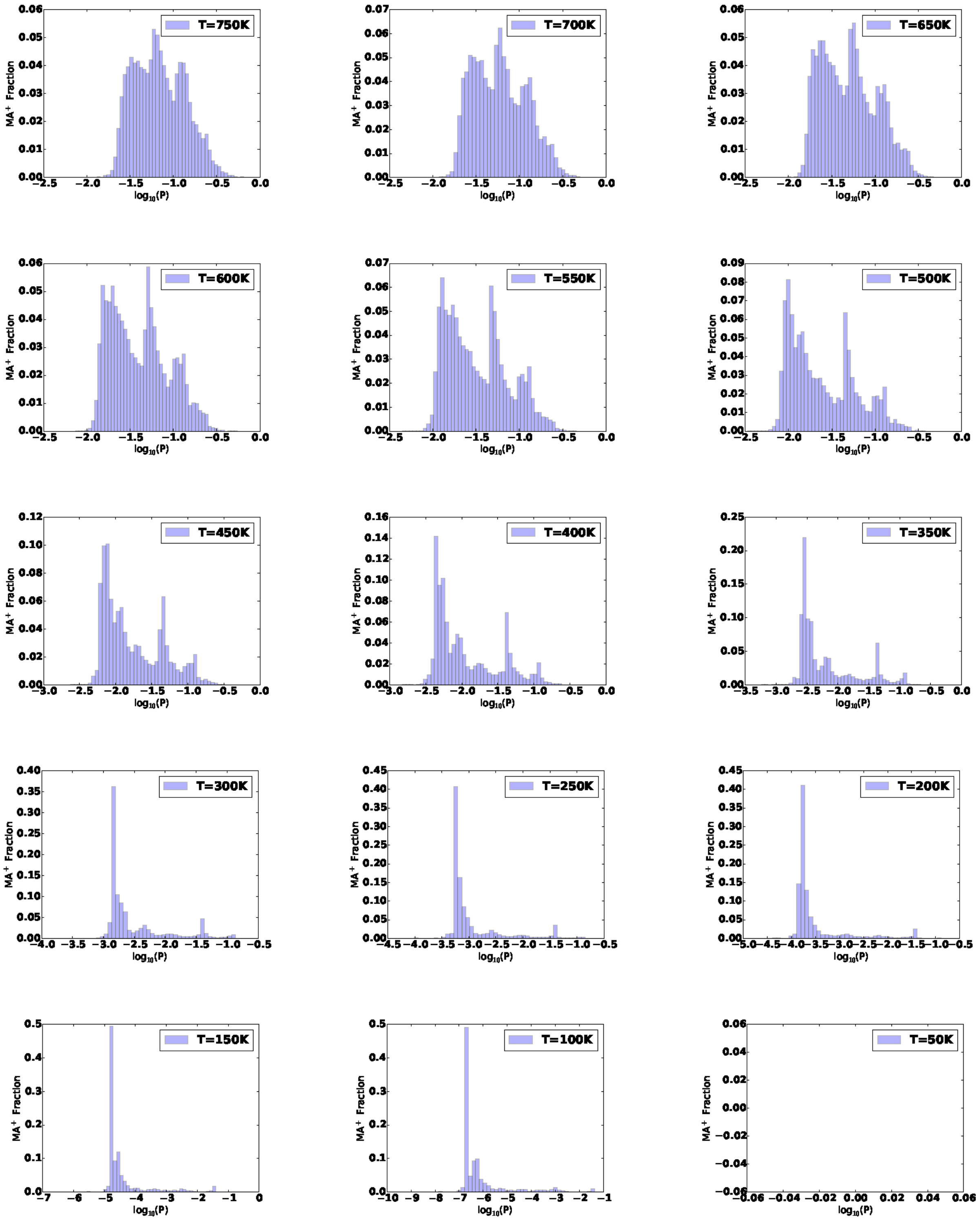

Supplementary Fig. 23. MA^+ reorientation probability distribution during cooling: ferroelectric case. Histograms from the full simulation lattice described in **Supplementary Fig. 20** for K = 100 meV showing the probability that MA^+ ions will undergo realignment on their next move trial. The temperatures indicated range from T = 750 K (top left) to T = 50 K (bottom right).

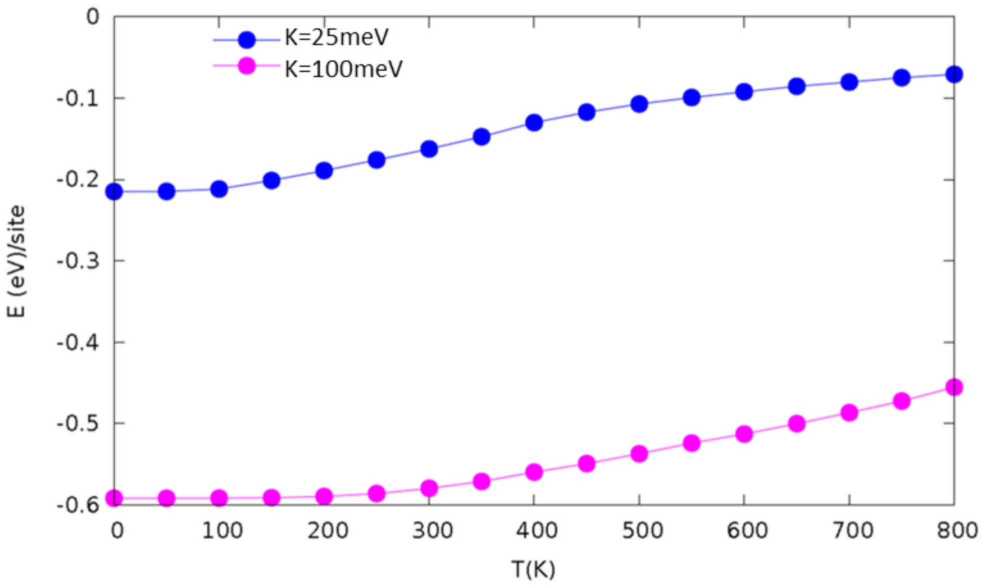

Supplementary Fig. 24. Internal energy of lattice during cooling. Plotted for both the *K* = 25 meV and K = 100 meV.

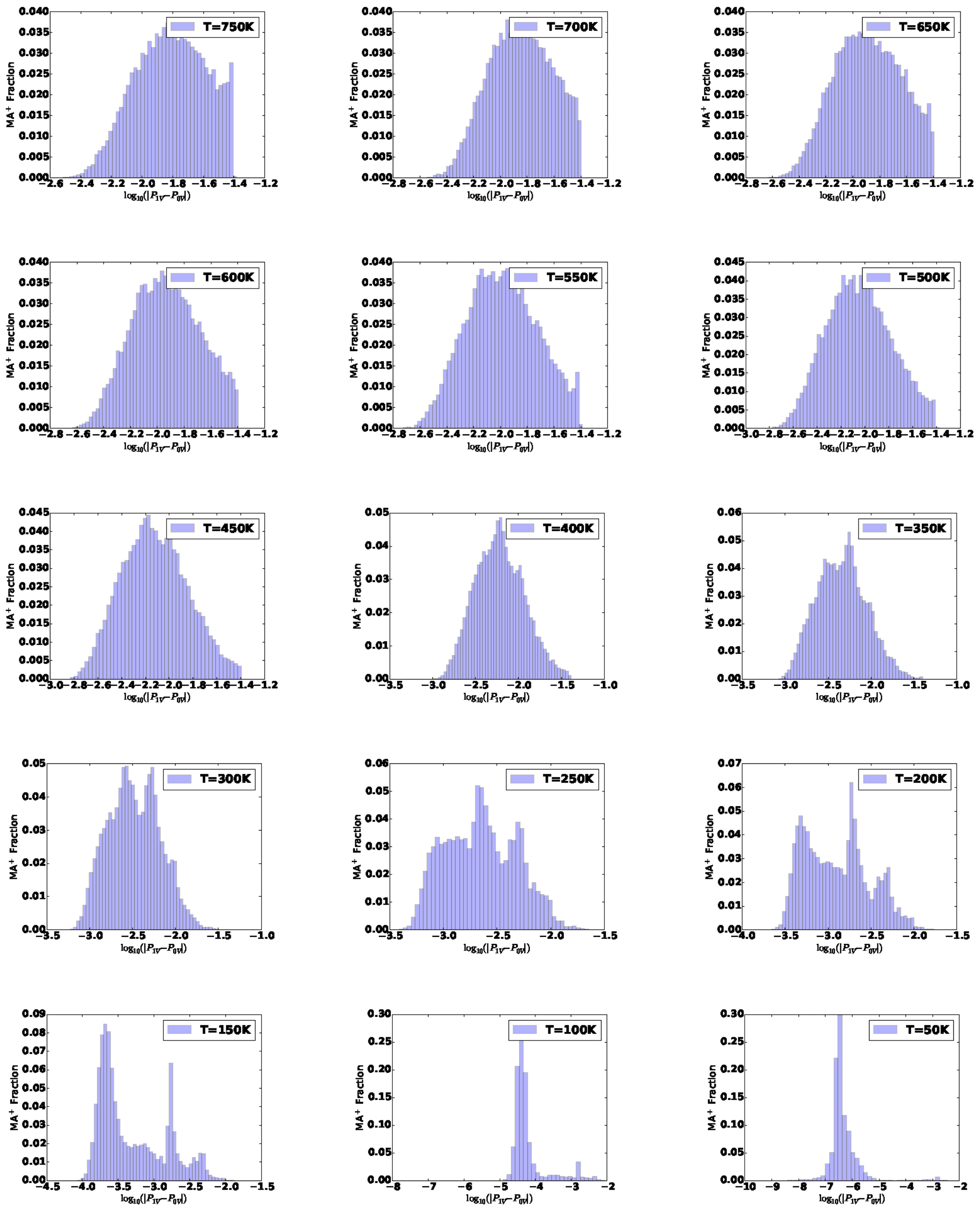

Supplementary Fig. 25. Effect of E-field on MA^+ reorientation probability distribution: antiferroelectric case. Histogram of the change in reorientation move probabilities, $|P_{1V} - P_{0V}|$, on application of an electric field (1 V corresponding to 5 MV m^{-1}) to a lattice with the orientations shown in **Supplementary Fig. 16**, K = 25 meV.

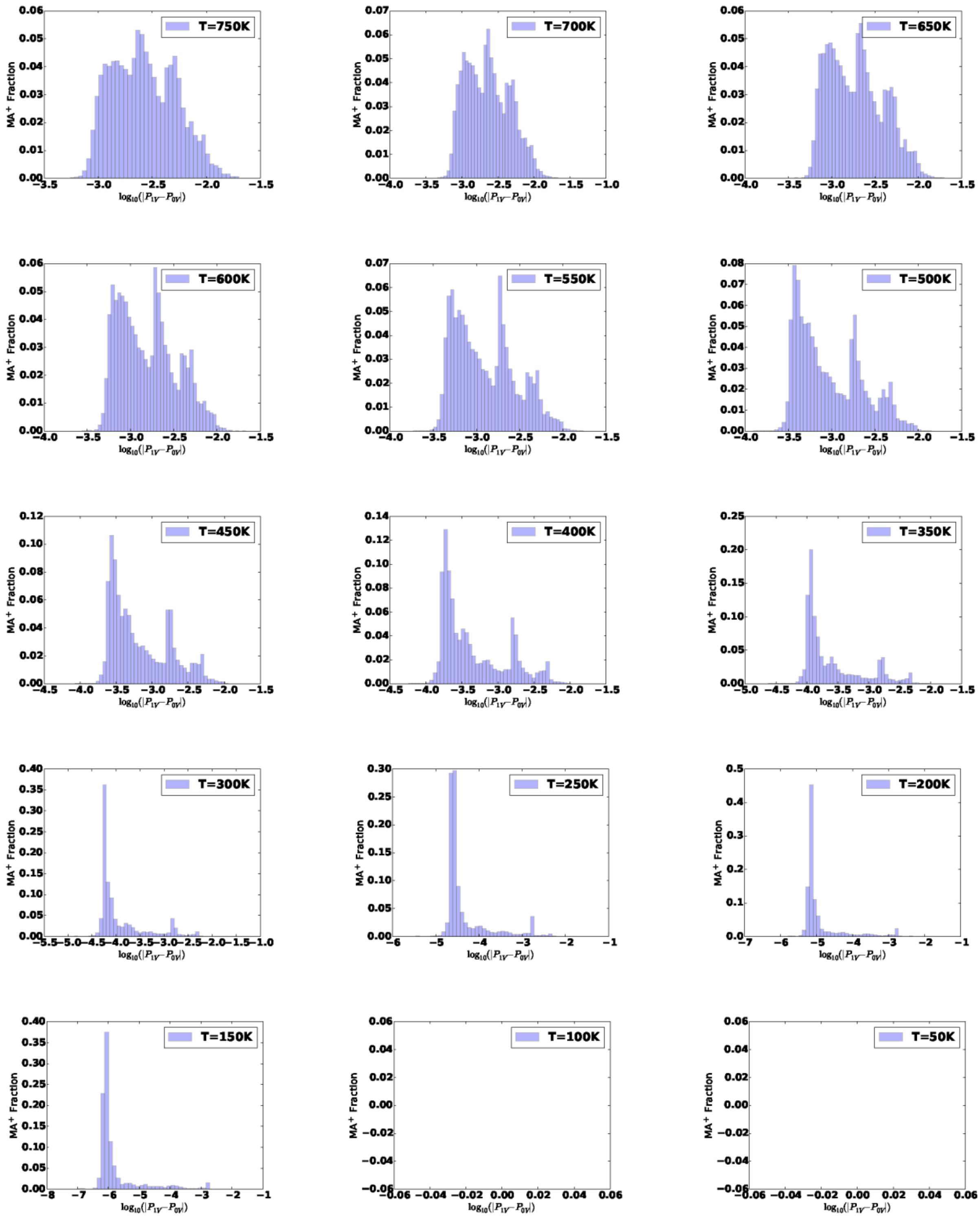

Supplementary Fig. 26. Effect of E-field on MA^+ reorientation probability distribution: ferroelectric case. Histogram of the change in reorientation move probabilities, $|P_{1V} - P_{0V}|$, on application of an electric field (1V corresponding to 5 MV m^{-1}) to a lattice with the orientations shown in **Supplementary Fig. 20**, K = 100 meV.

Supplementary Table 1. Terms to fit the scattering data, $R_i(Q)$. Coefficients for rotational models (see **Supplementary Note 2** and Equation 4, main text) of the EISF indicating the realignment type for rotational jumps between *N* equivalent sites.[1] The trajectory of the path between equivalent sites is given with the corresponding expression for $a_0(Q)$ as used in $S_{inc}(Q,\omega)$ where j_0 is a Bessel function of the first kind and order 0. The radii of rotational reorientation used in the model, *r*, is given, calculated from the geometry of the MA^+ ion and the path traced by the hydrogen nuclei for moves assigned to both mode '1' and mode '2' in **Fig. 1b**. Models labelled in **Fig. 3** of the main text are also indicated.

Realignment type	*N*	path between sites	$a_0(Q)$	*r* [Å]	label in **Fig. 3**
**Model for the rotation around the C-N axis (mode '1')**					
Methyl and ammonium 3-sites-rotation	3	conical	$\frac{1}{3}\left[1+2j_0(Qr\sqrt{3})\right]$	1.033 (CH_3)<br>0.958(NH_3)	
Methyl and ammonium 6-sites-rotation	6	conical	$\frac{1}{6}\left[1+2j_0(Qr)+2j_0(Qr\sqrt{3})+j_0(2Qr)\right]$	1.033 (CH_3)<br>0.958(NH_3)	
**Model for the rotation of the C-N axis (mode '2')**					
π-flips	2	direct	$\frac{1}{2}\left[1+j_0(2Qr)\right]$	1.697	(ii)
face-to-face	3	conical	$\frac{1}{3}\left[1+2j_0(Qr\sqrt{3})\right]$	1.386	(v)
edge-to-edge	4	direct	$\frac{1}{4}\left[1+2j_0(Qr\sqrt{2})+j_0(2Qr)\right]$	1.386	(iv)
face-to-face	4	direct	$\frac{1}{4}\left[1+2j_0(Qr\sqrt{2})+j_0(2Qr)\right]$	1.697	
edge-to-edge	4	conical	$\frac{1}{4}\left[1+2j_0(Qr\sqrt{2})+j_0(2Qr)\right]$	1.200	
corner-to-corner	4	conical	$\frac{1}{4}\left[1+2j_0(Qr\sqrt{2})+j_0(2Qr)\right]$	1.386	(iii)
free rotation			$j_0^2(Qr)$	1.697	(i)

Supplementary Table 2. Mean residence time of the hydrogen atoms involved in the rotations. Values determined for the temperatures with high statistics measurements only. Column 2 concerns the small radius rotation (methyl-rotor), while column 3 is for the reorientation of the C-N axis itself. The residence time (τ_i) of each mode can be inferred from the FWHM of their corresponding Lorenzian: $\tau_i = 2\hbar/\mathrm{FWMH}$.

Temperature in K	τ_1 (CH_3 and/or NH_3) [ps]	τ_2 (C-N) [ps]
370	2.6	12.7
300	3.1	13.8
250	3.3	13.7
180	3.1	27
140	8	23

Supplementary Note 1: The measured signal

Each of the forty-two detectors constituting the spectrometer records neutron counts per second, $I(Q,\omega)$ for a given detector angle (corresponding to momentum transfer Q) and change in scattered neutron energy ($\hbar\omega$). Since the energy exchanged is small, the change in momentum wavenumber Q is well approximated by the elastic $Q = 4\pi/\lambda \sin(\vartheta/2)$, where ϑ is the scattering angle. This scattering intensity is related to the double differential cross-section (σ) of the sample, that is, the probability that a neutron is scattered in a solid angle $d\Omega$ with an energy change dE (ϑ). This in turn depends on the scattering characteristics of the material which is dominated by incoherent scattering from hydrogen in our case and thus described by the incoherent scattering law, S_{inc}:[1,11,12]

$$I(Q,\omega) = \phi N \frac{\partial^2\sigma}{\partial\Omega\partial E} = \phi N \frac{k}{4\pi\hbar k_0}\sigma_{inc} S_{inc}(Q,\omega) \tag{1}$$

where N is the number of scatters (assumed to be hydrogen nuclei) in the beam, k is the wavenumber of the scattered neutrons, k_0 the wavenumber of the incident neutrons, and σ_{inc} is the incoherent scattering cross-section of the scatters. S_{inc} is a self-correlation function of nuclei at different times, which characterizes a particular type of motion.

Supplementary Note 2: Mathematical expressions of the models employed to fit the elastic to elastic and quasielastic ratio, $\boldsymbol{R_i(Q)}$

The general scattering Law for one motion can be written in the following form:[1]

$$S_{inc}(Q,\omega) = A_0(Q)\delta(\omega) + \sum_{l=1}^{N-1} A_l(Q) L_l(\omega) \tag{2}$$

where

$$A_0(Q) = a_0(Q) e^{-\langle u^2\rangle Q^2} \tag{3}$$

$$A_l(Q) = \frac{e^{-\langle u^2\rangle Q^2}}{N} \sum_{k=1}^{N} j_0\left(2Qr\sin\left(\frac{l\pi}{N}\right)\right)\cos\left(\frac{2kl\pi}{N}\right) \tag{4}$$

and

$$L_i(\omega) = \frac{1}{\pi}\frac{\tau_i}{1+\omega^2\tau_i} \tag{5}$$

δ is Dirac's delta function and j_0 the Bessel function of the first kind and order 0. The model assumes uniaxial jumps between N equivalent sites, equally spaced on a circle of radius r. The term $a_0(Q)$ is the elastic incoherent structure factor (EISF), which is non-vanishing for scatterers restricted to a delimited volume (i.e. short range diffusion or rotation).

Supplementary Table 1 gives the expressions for $a_0(Q)$ and parameter values used in the functional form of the EISFs models (Equation (4), main text) of the reorientations of the C-N axis (rotational mode '2') that were investigated as a function of Q. We note that due to the relatively minor changes in the lattice parameters of the MAPI cage across phase transitions, the geometry of these rotational models is not significantly influenced by the crystalline phase.[13]

Supplementary References

1 Bee, M. *Quasielastic Neutron Scattering, Principles and Applications in Solid State Chemistry, Biology and Materials Science*. (1988).

2 Volino, F. & Dianoux, A. J. Neutron incoherent scattering law for diffusion in a potential of spherical symmetry: general formalism and application to diffusion inside a sphere. *Molecular Physics* **41**, 271 - 279 (1980).

3 Dianoux, A. J., Pineri, M. & Volino, F. Neutron incoherent scattering law for restricted diffusion inside a volume with an anisotropic shape. *Molecular Physics* **46**, 129-137 (1982).

4 Pellet, N., Teuscher, J., Maier, J. & Graetzel, M. Transforming Hybrid Organic Inorganic Perovskites by Rapid Halide Exchange. *Chemistry of Materials*, Article ASAP (2015).

5 *Aldrich library of infrared spectra*. (Aldrich Chemical Company, 1981).

6 Jeon, N. J. *et al.* Solvent engineering for high-performance inorganic–organic hybrid perovskite solar cells. *Nat Mater* **13**, 897-903, doi:10.1038/nmat4014 (2014).

7 Mosconi, E., Quarti, C., Ivanovska, T., Ruani, G. & De Angelis, F. Structural and electronic properties of organo-halide lead perovskite: a combined IR-spectroscopy and ab initio molecular dynamics investigation. *Phys. Chem. Chem. Phys.* **16**, 16131-16144 (2014).

8 Hannon, A. C. Results on disordered materials from the GEneral Materials diffractometer, GEM, at ISIS. *Nuclear Instruments and Methods in Physics Research A* **551**, 88-107 (2005).

9 Prager, M., Dupree, K.-H. & Mueller-Warmuth, W. Rotational Tunnelling and Methyl Group Reorientation in $Sn(CH_3)_4$ by Inelastic Neutron Scattering and Nuclear Magnetic Resonance. *Condensed Matter* **51**, 309-318 (1983).

10 Chahid, A., Alegria, A. & Colmenero, J. Methyl-group Dynamics in Ploly(vinyl methyl ether). A Rotation Rate Distribution Model. *Macromolecules* **27**, 3282-3288 (1994).

11 Dianoux, A. J. *Quasi-Elastic and Inelastic Neutron Scattering*. (Springer, 1988).

12 Higgins, J. S. & Benoit, H. C. *Polymers and Neutron Scattering*. (Clarendon Press, 1997).

13 Mashiyama, H., Kurihara, Y. & Azetsu, T. Disordered Cubic Perovskite Structure of $CH_3NH_3PbI_3$ (X=Cl, Br, I). *Journal of the Korean Physical Society* **32**, 156-158 (1998).
